# Loss of p53-inducible long non-coding RNA *LINC01021* increases chemosensitivity

**DOI:** 10.18632/oncotarget.22245

**Published:** 2017-11-01

**Authors:** Markus Kaller, Ursula Götz, Heiko Hermeking

**Affiliations:** ^1^ Experimental and Molecular Pathology, Institute of Pathology, Ludwig-Maximilians-University Munich, Munich, Germany; ^2^ German Cancer Consortium (DKTK), Heidelberg, Germany; ^3^ German Cancer Research Center (DKFZ), Heidelberg, Germany

**Keywords:** p53, LINC01021, chemosensitivity, colorectal cancer, tumor suppression

## Abstract

We have previously identified the long non-coding RNA *LINC01021* as a direct p53 target (Hünten et al. Mol Cell Proteomics. 2015; 14:2609-2629). Here, we show that *LINC01021* is up-regulated in colorectal cancer (CRC) cell lines upon various p53-activating treatments. The *LINC01021* promoter and the p53 binding site lie within a MER61C LTR, which originated from insertion of endogenous retrovirus 1 (ERV1) sequences. Deletion of this MER61C element by a CRISPR/Cas9 approach, as well as siRNA-mediated knockdown of *LINC01021* RNA significantly enhanced the sensitivity of the CRC cell line HCT116 towards the chemotherapeutic drugs doxorubicin and 5-FU, suggesting that *LINC01021* is an integral part of the p53-mediated response to DNA damage. Inactivation of *LINC01021* and also its ectopic expression did not affect p53 protein expression and transcriptional activity, implying that *LINC01021* does not feedback to p53. Furthermore, in CRC patient samples *LINC01021* expression positively correlated with a wild-type p53-associated gene expression signature. *LINC01021* expression was increased in primary colorectal tumors and displayed a bimodal distribution that was particularly pronounced in the mesenchymal CMS4 consensus molecular subtype of CRCs. CMS4 tumors with low *LINC01021* expression were associated with poor patient survival. Our results suggest that the genomic redistribution of ERV1-derived p53 response elements and generation of novel p53-inducible lncRNA-encoding genes was selected for during primate evolution as integral part of the cellular response to various forms of genotoxic stress.

## INTRODUCTION

The p53 transcription factor is encoded by a tumor suppressor gene, which represents the most commonly mutated gene in human cancer [1]. In addition, many of the cancers without p53 mutation harbor alterations up- or down-stream of p53, which also impede the ability of p53 to suppress tumors. P53 and its loss may represent attractive targets for tumor therapy [2]. Most *p53* mutations target the DNA binding properties of p53, suggesting that the regulation of specific target genes is central for the tumor suppression mediated by p53. The transcriptional activity of p53 is induced by various forms of cellular stress, such as DNA damage or aberrant oncogene activation [3]. P53 directly activates a large set of genes, which mediate numerous cellular functions involved in tumor suppression, such as cell cycle arrest, apoptosis, senescence, and DNA repair [3]. Apart from protein-coding genes, non-coding RNAs are transcriptional targets of p53. MicroRNAs (miRNAs) have been extensively characterized as important mediators for down-regulation of mRNA and protein expression caused by p53, thereby inhibiting pro-tumorigenic processes, such as proliferation, stemness, and epithelial-mesenchymal transition (EMT) [4]. More recently, long non-coding RNAs (lncRNAs) have emerged as downstream effectors of tumor suppression by p53 (reviewed in [5, 6]).

LncRNAs have been defined by a length of >200 nucleotides which distinguishes them from small non-coding RNAs, such as miRNAs. Recent analyses indicate that the human genome encodes for approximately 32.000 lncRNAs, which therefore represent a large class of transcripts, comparable in number to protein-coding mRNAs [7]. However, the function and biological relevance of the large majority of these transcripts is not well understood. Moreover, compared to miRNAs their functions appear to be more diverse, ranging from regulation of chromatin structure, sequestration of microRNAs as so-called competitive endogenous RNAs (ceRNAs) to regulation of mRNA stability, processing or translation, and the modulation of protein–protein interactions [7-10]. Growing evidence points to a role of the deregulation of certain lncRNAs in the etiology of a number of diseases, among them cancer.

Several genome-wide studies in murine and human cells have identified numerous lncRNAs that are direct p53 target genes [11-16], and a growing number of these lncRNAs have important roles in the p53 transcriptional network as both positive and negative regulators of p53 function (reviewed in [5, 6]). For example, *lincRNA-p21* is driven from a bidirectional promoter also regulating the *CDKN1A* gene (encoding the p21 protein) and is involved both in transcriptional and post-transcriptional repression of specific genes via association with the hnRNPK protein and the RNA-binding protein HuR [13, 17]. More recently, the *DINO* (DNA damage induced noncoding) lncRNA, which is also located upstream of the *CDKN1A* gene and activated in a p53 dependent manner, was shown to regulate expression of p53 target genes by binding and stabilization of the p53 protein [18]. Conversely, *NEAT1*/*AP000944.1* has been characterized as a direct p53 target that attenuates p53 activation by modulating ATR signaling in response to DNA damage [19].

Transposable elements (TEs) are a major source for regulatory sequences in the vertebrate genome [20, 21], and transcription of a substantial fraction of lncRNAs is induced by TEs [22, 23]. Moreover, exonic sequences of lncRNAs often are derived from TE sequence insertions into the genome, and some of these lncRNAs are specifically activated in various types of cancer [22, 24, 25]. Evolutionarily, p53 has been implicated in the control of mobile elements such as retrotransposons [26], and p53 has been shown to bind to repetetive elements [27] and mediate their epigenetic silencing [28]. Counterintuitively, at least some subfamilies of TE long terminal repeats (LTRs) have been reported to harbor functional p53 response elements involved in transcriptional activation. The MER61C and LTR10D LTRs, which are derived from endogenous retrovirus 1 (ERV1) sequences, that invaded the primate lineage 40-63 million years ago, display strong enrichment for predicted p53 binding sites [29]. About 53% of all MER61C elements (157/294) in the human genome harbor a predicted p53 binding site, and a small number of these elements has been characterized as enhancers/promoters required for p53-dependent activation of adjacent genes [29]. Interestingly, phylogenetic analyses of MER61C elements showed that the p53 binding sites were already present upon genomic integration, suggesting the introduction of substantial numbers of novel p53 response elements into the genome during primate evolution via this route. However, the functional significance of these ERV1-derived p53 binding sites and their impact on p53-regulated cellular processes and underlying transcriptional programs have remained largely unexplored.

We previously determined the genome-wide DNA binding pattern of p53 by a ChIP-Seq analysis and detected differential lncRNA expression after activation of a conditional *p53* allele using RNA-Seq in SW480 CRC cells [30]. Thereby, we identified several lncRNAs as novel, direct p53 target genes. Among them *LINC01021* (also designated *LOC643401* or *RP11-46C20.1*) was one of the lncRNAs most strongly activated by p53.

Here, we show that p53-dependent transcription of *LINC01021* is driven by a MER61C LTR, which represents a remnant of an ERV1 integration event. Furthermore, the *LINC01021* genomic locus harbors additional remnants of ERV1 DNA within its exonic and intronic sequences, as well as additional repetitive DNA elements. Ablation of *LINC01021* transcription in p53-proficient colorectal cancer cells by CRISPR/Cas9-mediated deletion of the MER61C LTR results in hypersensitivity towards chemotherapeutic treatments. Our results suggest that *LINC01021* contributes to cellular survival in response to genotoxic stress by suppressing apoptosis.

## RESULTS

### *LINC01021* is a direct p53 target regulated by an ERV1-derived LTR and its expression is highly dependent on p53 in CRC cell lines

**Figure 1:**
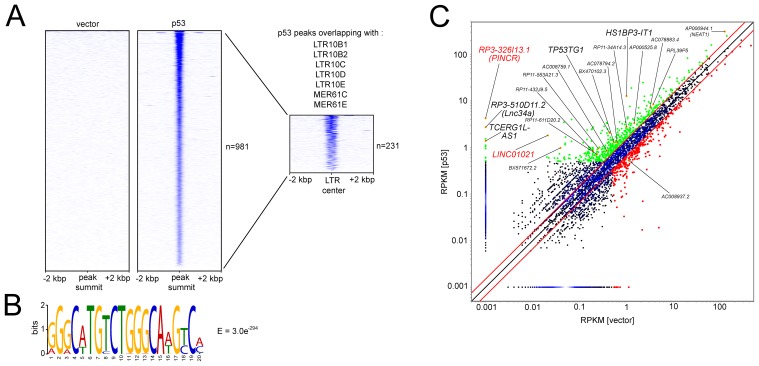
Identification of ERV1 LTR elements within promoters of p53-inducible lncRNAs **(A)** Heatmap visualization depicting p53 binding events ranked by read number in a 4 kbp window up- and down-stream of high-confidence p53 ChIP-Seq peaks identified with MACS2 peak calling after ectopic expression of p53 or vector control in SW480 cells [30]. P53-bound regions were filtered for regions intersecting with genomic locations of the indicated ERV1-derived LTR sequences. Genomic coordinates for 1839 LTR sequences were obtained from the UCSC genome browser. The resulting heatmap visualization depicts p53 binding ranked by read number in a 4 kbp window up- and downstream of the LTR center. **(B)** Genomic DNA sequences associated with p53-bound LTRs (n=231) were subjected to MEME analysis. The resulting p53 DNA binding motif and statistical significance (E-value) are indicated. **(C)** Scatter plot representation of RNA-Seq data obtained and adapted from our NGS analysis previously published in Hünten et al. [30] showing differential lncRNA expression after induction of p53 in SW480 cells. Red lines denote 1.5-fold expression change cut-offs. LncRNAs with ≥ 1.5-fold upregulation and RPKM > 0.5 in at least one condition are shown in green, LncRNAs with ≥ 1.5-fold downregulation and RPKM > 0.5 in at least one condition shown in red. Orange dots indicate lncRNAs with p53 binding within +/- 20 kbp of the TSS. LncRNAs with ERV1-associated TSSs are highlighted in red, additional lncRNAs without ERV1-associated TSSs in black. *RP3-326I13.1/PINCR*, *RP3-510D11.2/Lnc34a*, *TP53TG1*, and *NEAT1/ AP000944.1* have been functionally characterized recently [19, 48, 56, 57].

**Figure 2:**
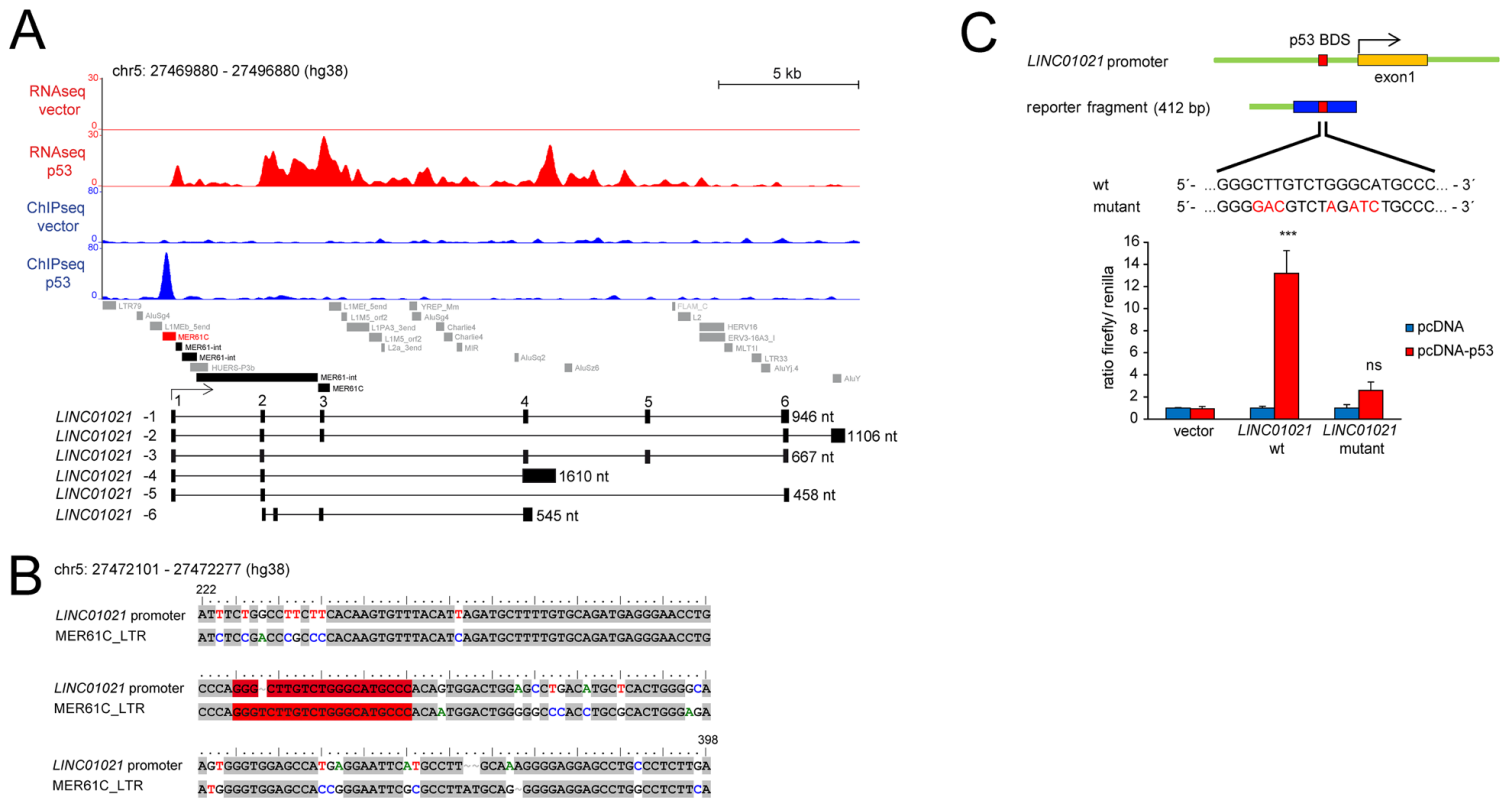
Analysis of p53-binding and retroviral elements within the *LINC01021* promoter region **(A)** RNA-Seq and ChIP-Seq results were obtained after induction of ectopic *p53* in SW480 cells and adapted from our previous NGS analysis published in Hünten et al. [30]. Genomic coordinates of repetitive DNA elements were obtained by analyzing the DNA sequence of the indicated genomic interval using the Dfam database [33]. The MER61C element harboring the p53 binding site associated with the *LINC01021* promoter is indicated in red. Additional MER61 DNA sequences are shown in black, other repetitive elements in grey. Lower part: Schematic representation of the *LINC01021* genomic locus and its annotated transcript variants. The length of spliced transcripts is indicated. **(B)** Alignment of the *LINC01021* promoter sequence analyzed in luciferase reporter assays and the MER61C profile hidden Markov model (HMM) obtained from the Dfam database. The p53 binding site is given in red. Pairwise sequence alignment was performed with Promoterwise [58] and edited with the BioEdit software. Only the proximal promoter sequence with sequence homology to MER61C is shown. The nucleotide positions within the reporter fragment used for luciferase reporter assays are indicated. **(C)** A 412 bp fragment of the *LINC01021* promoter harboring the p53 binding site or a mutated version was cloned upstream of the luciferase coding sequence. The proximal promoter sequence with sequence homology to MER61C is high-lighted in blue. Base exchanges in the p53 binding site of the mutated promoter fragment are highlighted in red. RKO *p53-/-* cells were transfected with the indicated reporter constructs and subjected to dual reporter assays 48 hours post transfection. Renilla activity was used for normalization. Results are represented as mean +/- s.d. (*n*=3).

We previously determined the genome-wide DNA binding pattern of p53 by a ChIP-Seq analysis and detected differential lncRNA expression after activation of a conditional *p53* allele using RNA-Seq in SW480 CRC cells [30]. Several studies have shown that many p53-bound genomic regions represent repetitive DNA elements [27, 28, 31]. ERV1-derived LTRs of several MER61 and LTR10 subfamilies are highly enriched for potential p53 binding sites, and some of them function as *bona fide* p53 response elements driving the expression of adjacent protein-coding genes [29]. Here we found that 231 of 981 high-confidence (FDR q-value < 0.05) p53 ChIP-Seq peaks identified in SW480 cells are localized within LTRs of MER61 and LTR10 subfamilies (Figure 1A). Furthermore, MEME analysis [32] identified the p53 consensus binding sequence within the DNA sequences associated with these p53-bound LTRs (Figure 1B). Next, we analyzed which of the p53-bound ERV1-derived LTRs are located in the vicinity (+/- 5 kbp) of the transcription start sites (TSSs) of the p53-induced lncRNAs we had previously identified. Indeed, the TSSs of two lncRNAs that were strongly up-regulated upon induction of p53 in the SW480 cell line, *RP3-326I13.1* and *LINC01021*, were located in close proximity to an ERV1-derived MER61C LTR (Figure 1C). Analysis of the *LINC01021* genomic locus using the Repeatmasker software and the Dfam database [33] for the presence of repetitive DNA elements confirmed that the proximal promoter region bound by p53 is composed of a MER61C element (Figure 2A). Moreover, the entire 5´-region of the *LINC01021* genomic locus is composed of DNA sequences derived from both LTR and internal regions of the MER61 subfamily of ERV1 (Figure 2A). The ENSEMBL genome annotation for *ENSG00000250337/LINC01021* includes six alternatively spliced transcripts and the exons 1-3 of isoforms 1-5 are located within remnants of ERV1-derived DNA (Figure 2A), indicating that a substantial portion of the *LINC01021* genomic locus is derived from the integration of repetitive DNA elements which confer either p53 responsiveness and/or contribute to *LINC01021* exon sequences.

In order to determine whether the MER61C sequence localized upstream of the *LINC01021* TSS is sufficient to mediate p53-dependent activation of *LINC01021* transcription, we inserted a 412 bp fragment (Figure 2B) including the p53 response element into a reporter plasmid upstream of a luciferase open reading frame. Dual reporter luciferase assays showed that this reporter fragment is indeed able to mediate induction of luciferase expression upon co-expression of ectopic p53 (Figure 2C). Moreover, mutations at several nucleotide positions within the p53 binding site largely abolished the inducibility by p53. Therefore, the MER61C LTR sequence upstream of the *LINC01021* TSS is sufficient for mediating p53-responsiveness.

**Figure 3:**
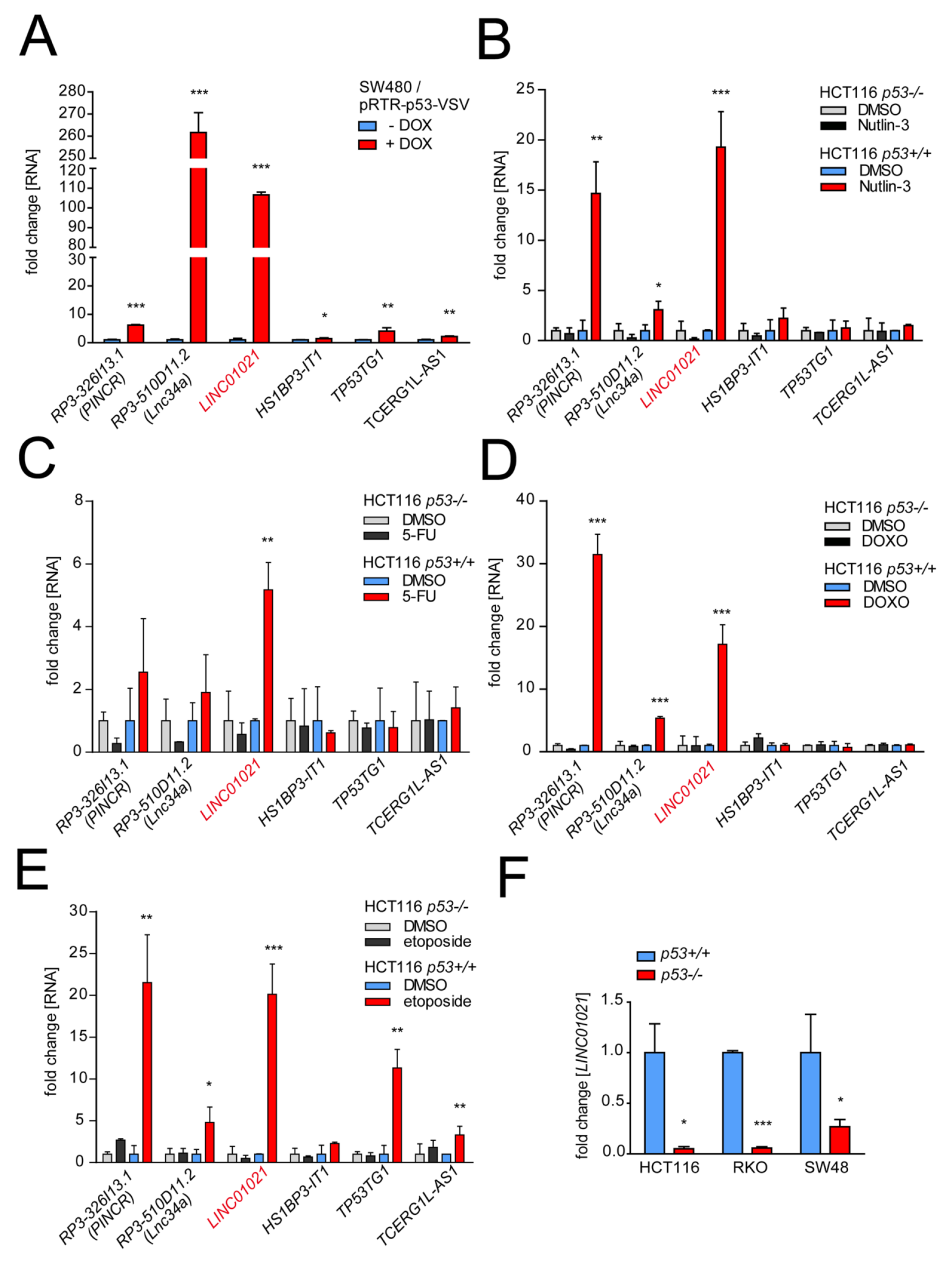
Comparative analysis of lncRNA regulation by p53 in CRC cell lines **(A)** Expression of selected lncRNAs was determined by qPCR analyses after induction of ectopic p53 by addition of doxyxcycline (DOX) in SW480 cells for 24 hours and normalized to the expression in untreated cells and to *GAPDH*. **(B-E)** Expression of selected lncRNAs was determined by qPCR analyses after treatment of HCT116 *p53*+/+ and *p53*-/- cell lines with 5-FU, doxorubicin (DOXO), etoposide, Nutlin-3 or, as a control, DMSO for 24 hours, and normalized to the expression in untreated cells and to *GAPDH*. **(F)** *LINC01021* expression was analyzed by qPCR in isogenic *p53*+/+ and *p53*-/- HCT116, RKO and SW48 cell lines and normalized to the expression in *p53*+/+ cells and to *GAPDH*. (A-F) Results are represented as mean +/- s.d. (*n*=3).

Intriguingly, the promoter of *RP3-326I.13/PINCR*, which we previously identified as a direct p53 target gene [30], also contains a MER61C LTR with high sequence similarity to the element described here for the *LINC01021* promoter (Supplementary Figure 1). In order to compare ERV1 LTR-driven expression of lncRNAs *LINC01021* and *RP3-326I13.1/PINCR* to that of other p53-inducible lncRNA-encoding genes (depicted in Figure 1C) that do not contain ERV1-derived promoter elements, we determined their expression after activation of p53. First, we validated induction of selected lncRNAs in SW480 cells after activation of a conditional *p53* allele (Figure 3A). Next, we subjected the CRC cell line HCT116 harboring wild-type *p53* (HCT116 *p53*+/+) and *p53*-deficient HCT116 cells (HCT116 *p53*-/-) to treatment with the MDM2 inhibitor Nutlin-3 or the chemotherapeutic agents etoposide, doxorubicin (DOXO) and 5-fluorouracil (5-FU; Figure 3B-3E). Whereas expression of some of the selected lncRNAs did not increase significantly upon treatment or showed only modest increases, *LINC01021* displayed consistent and strong up-regulation in a p53-dependent manner after all treatments. Furthermore, basal *LINC01021* expression was significantly reduced in *p53*-deficient derivatives of the *p53*-proficient HCT116, RKO and SW48 CRC cell lines (Figure 3F).

### CRISPR/Cas9-mediated deletion of the *LINC01021* promoter

**Figure 4:**
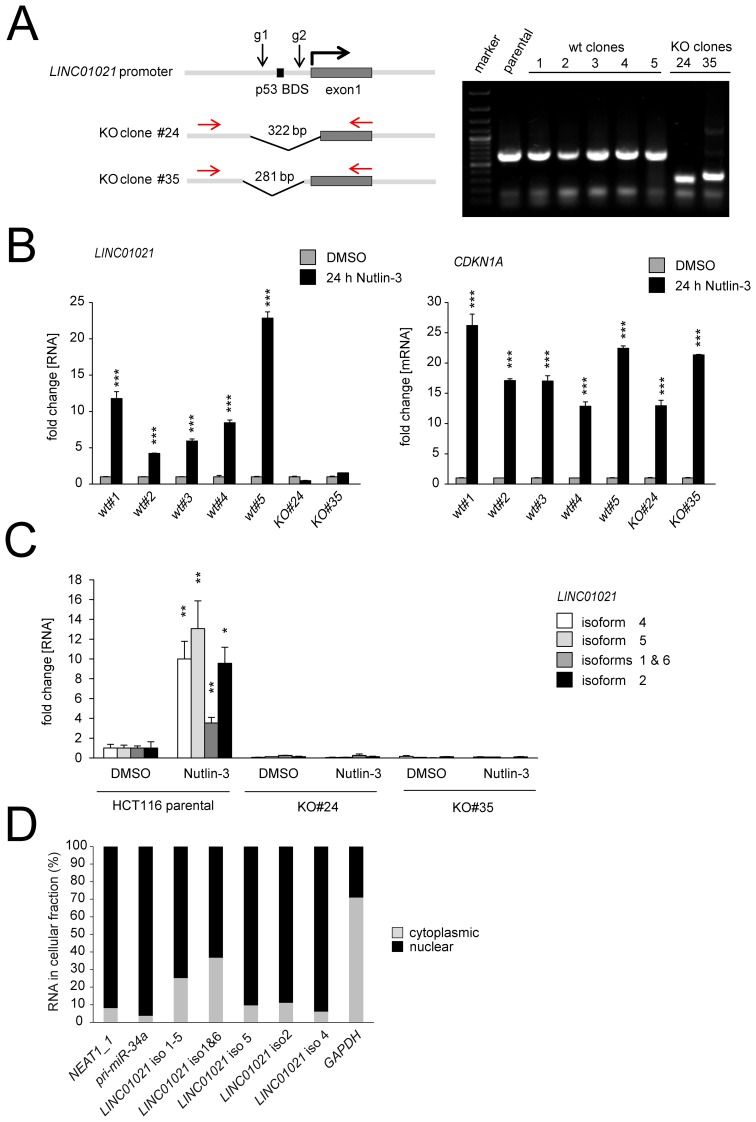
Characterization of *LINC01021* by CRISPR/Cas9-mediated deletion of the promoter elements, isoform-specific qPCR and subcellular fractionation **(A)** Position of guide RNAs (g1 and g2) used to delete portions of the *LINC01021* promoter. Genomic DNA from single cell HCT116 clones was analyzed by PCR with the indicated primers (red). **(B)** Expression of *LINC01021* was determined by qPCR after treatment with Nutlin-3 for 24 hours. *CDKN1A* served as a positive control for p53-mediated target gene induction. **(C)** Expression of *LINC01021* isoforms was verified by qPCR with isoform-specific primers in parental HCT116 cells and *LINC01021* KO clones. Expression was normalized to untreated, parental HCT116 cells and *GAPDH*. (A-C) Results are represented as mean +/- s.d. (*n*=3). **(D)** The subcellular localization of *LINC01021* splice variants was determined in HCT116 cells by qPCR analyses after fractionation of cytoplasmic and nuclear RNA as described [51]. *NEAT1_1* and *pri-miR-34a* were used as positive controls for nuclear RNAs, *GAPDH* was used as a positive control for cytoplasmic RNA localization.

Next, we intended to abrogate expression of all *LINC01021* transcript variants by CRISPR/Cas9-mediated deletion within the *LINC01021* promoter regions using two guide RNAs targeting regions within the MER61C sequence flanking the p53 binding site (Figure 4A). After two independent transfections of HCT116 cells with sgRNA-expression plasmids, FACS sorting of GFP-positive cells and expansion of single cell clones, we obtained two clones with deletions in the *LINC01021* promoter (Figure 4A, Supplementary Figure 2). As controls, we generated single-cell clones from HCT116 cells transfected with pSp-Cas9 plasmid harboring no sgRNAs (Figure 4A). Whereas KO clone #24 carries a deletion that extends beyond the promoter region and also includes the annotated transcription start site (TSS), as well as a significant portion of exon 1, KO clone #35 harbors a deletion that leaves the *LINC01021* TSS intact (Figure 4A, Supplementary Figure 2). The five different wild-type HCT116 single-cell clones obtained here displayed *LINC01021* induction upon p53 activation with Nutlin-3, whereas induction of *LINC01021* was completely abolished in both KO clones. Induction of the proto-typic p53 target gene *CDKN1A/p21* was not obviously affected by loss of *LINC01021* induction (Figure 4B). Moreover, qPCR-analysis of individual *LINC01021* isoforms showed that basal expression levels as well as p53-mediated induction of all tested *LINC01021* transcript variants was severely reduced in both KO clones compared to parental HCT116 cells. Therefore, the p53 response element is necessary for mediating basal *LINC01021* expression (Figure 4C).

Many lncRNAs predominantly localize to the nucleus and are associated with chromatin [34, 35]. Indeed, subcellular fractionation of HCT116 *p53+/+* cells followed by qPCR analysis of cytoplasmic and nuclear RNA fractions showed that all *LINC01021* transcript variants predominantly localize to the nucleus (Figure 4D), strongly suggesting a nuclear function for these lncRNAs.

### Loss of *LINC01021* causes hypersensitivity to chemotherapeutic treatments

**Figure 5:**
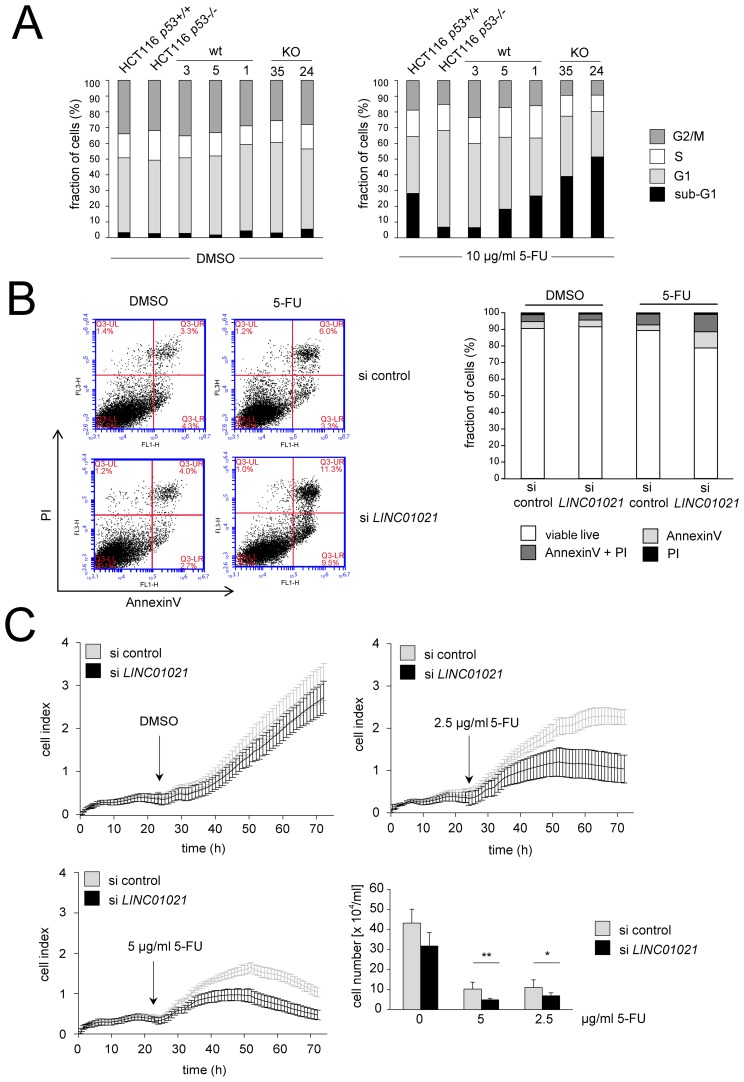
Loss of *LINC01021* sensitizes HCT116 cells to 5-FU **(A)** Parental HCT116, HCT116 *p53-/-* cells and *LINC0121* wt or KO clones were treated with DMSO or 5-FU at a final concentration of 10 μg/ml for 24 hours. After staining of DNA with propidium iodide samples were subjected to FACS analysis. **(B)** HCT116 cells were transfected with 40 nM siRNA pools consisting of 30 different siRNAs that target all *LINC01021* splice variants (siPOOLs) or control siPOOLs. 24 hours after transfection, 5-FU or DMSO was added. After 24 hours apoptosis was determined by FACS detection of Annexin-V positive cells. Left: representative dot plots. Right: quantification of biological replicates. Results are represented as mean +/- s.d. (n=3). **(C)** HCT116 cells were seeded at 1x10^4^ cells per E-plate well and transfected with 40 nM final siPOOL concentration. 24 hours after transfection, 5-FU or DMSO was added at the indicated final concentration. Cellular impedance was measured for 48 hours. For cell counting, cells from the same transfection were seeded at 1x10^4^ cells per 96-well and viable cells were enumerated 48 hours after addition of 5-FU at the indicated concentrations or DMSO using trypan blue exclusion. Cell numbers are displayed as mean +/- s.d. (*n*=3).

Next, we analyzed whether loss of the p53-inducible *LINC01021* affects the cellular response to chemotherapeutics, and treated *LINC01021* KO and wt clones, as well as the parental HCT116 *p53*+/+ cell line and its isogenic HCT116 *p53*-/- derivative with 5-FU and determined cell cycle distribution by DNA-content determination using FACS analyses. Interestingly, both *LINC01021* KO clones displayed higher proportions of apoptotic sub-G_1_ phase cells compared to wt clones and parental HCT116 cells after exposure to 5-FU (Figure 5A). Therefore, loss of *LINC01021* renders cells more sensitive to 5-FU-induced apoptosis.

In order to determine the role of *LINC01021* by an alternative approach, we down-regulated *LINC01021* expression in HCT116 cells using siRNA pools consisting of 30 individual siRNAs that target all *LINC01021* splice variants. Basal *LINC01021* expression in untreated HCT116 cells was slightly affected by transfection with *LINC01021*-specific siRNAs and was significantly reduced upon activation of p53 by either 5-FU or Nutlin-3 in combination with siRNA transfection (Supplementary Figure 3A, 3B). Knockdown of *LINC01021* did not affect expression of *CDKN1A*/*p21*, again indicating that p53 function was not affected by downregulation of *LINC01021* (Supplementary Figure 3A, 3B). Furthermore, expression of all *LINC01021* isoforms was significantly reduced by siRNA transfection to a similar extent upon p53 activation by Nutlin-3 (Supplementary Figure 3C).

**Figure 6:**
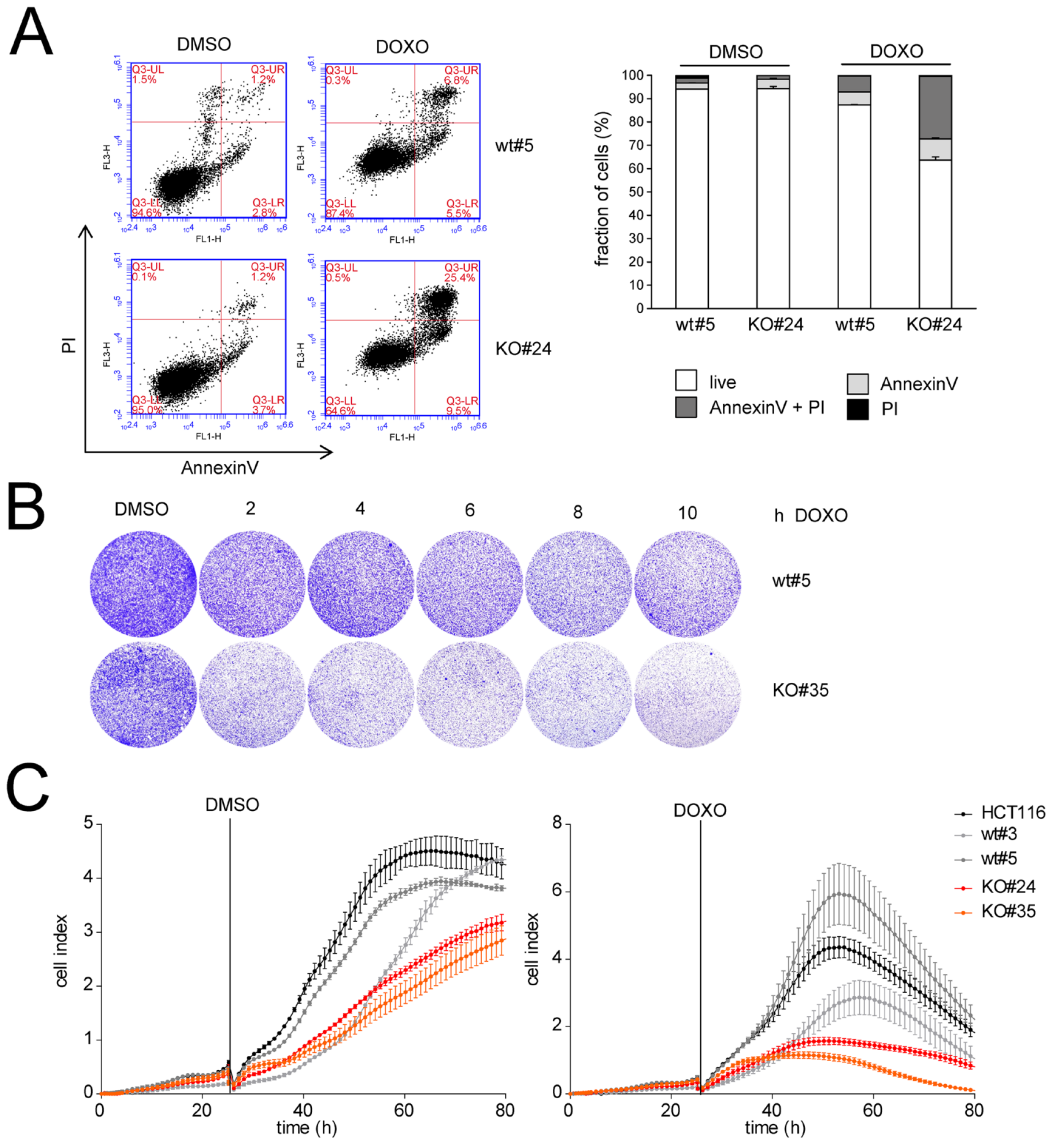
*LINC01021* inactivation sensitizes to doxorubicin **(A)** Apoptosis was determined by FACS analysis of Annexin-V/PI stained cells after treatment of *LINC01021* wt and KO clones with DMSO or doxorubicin (DOXO) for 40 hours. Left: representative FACS plots. Right: Quantification. **(B)** Colony formation assays of *LINC01021* wt and KO clones. Cells were seeded, cultivated for 24 hours and treated with DMSO or DOXO for the indicated periods. After removal of DOXO cells were incubated for 2 additional days in fresh medium before fixation and crystal violet staining. **(C)** Cellular impedance was determined after seeding 1x10^4^ cells per E-plate well and treatment with DMSO or DOXO after 25 hours. Values represent mean +/- s.d. (*n*=3).

**Figure 7:**
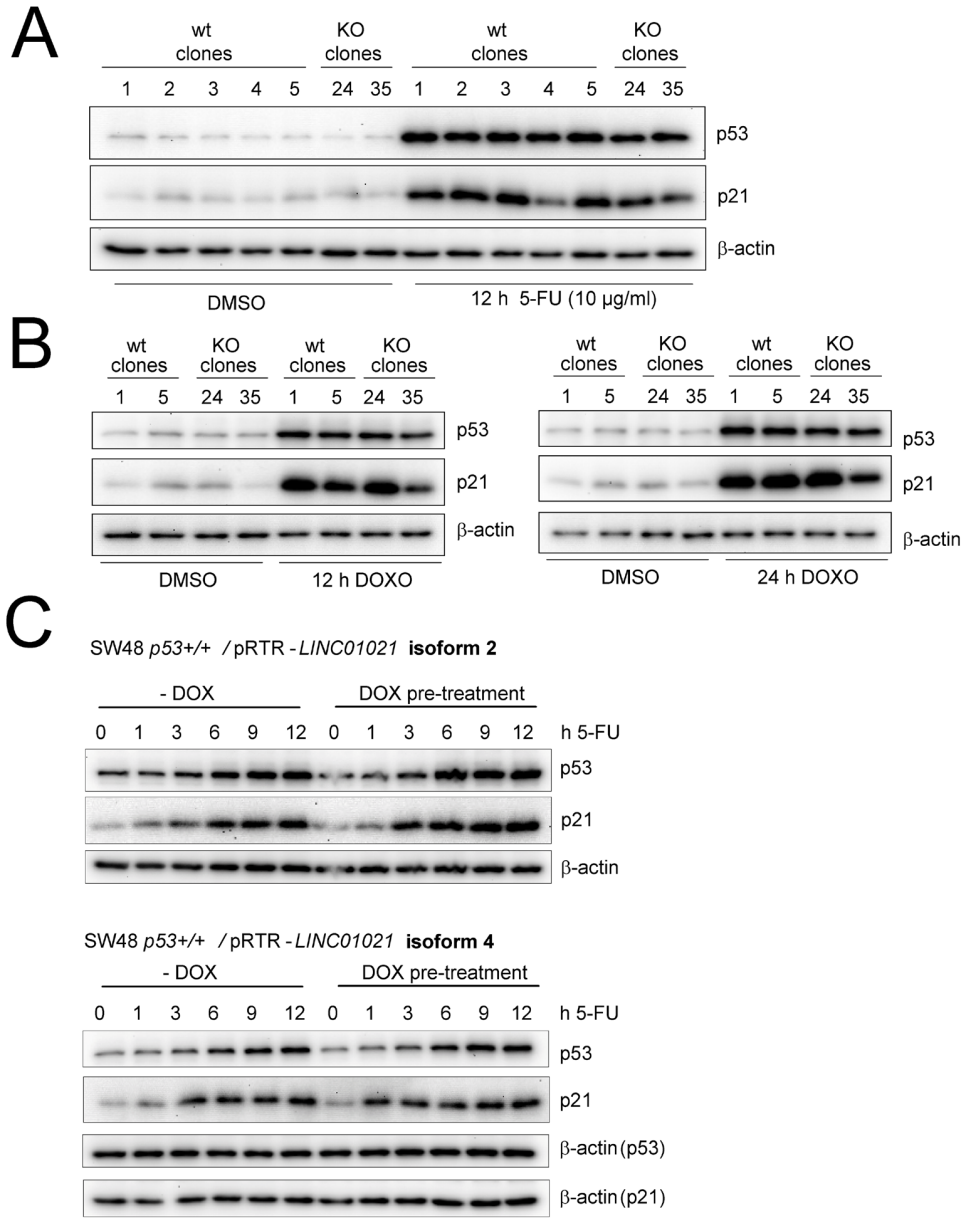
Modulating *LINC01021* expression does not affect p53 activation **(A)** Western blot analysis of p53 and p21 expression after treatment of *LINC01021* wt and KO HCT116 cell lines with DMSO or 5-FU for 12 hours. **(B)** Western blot analysis of p53 and p21 protein expression after treatment of *LINC01021* wt and KO HCT116 cell lines with DMSO or doxorubicin (DOXO) for 12 (left) or 24 hours (right). **(C)** Western Blot analysis of p53 and p21 expression after treatment of SW48 *p53+/+* expressing different *LINC01021* isoforms with DMSO or 5-FU for the indicated time periods. Ectopic *LINC01021* expression was induced by addition of doxycycline (DOX) for 32 hours (pre-treatment) before addition of 5-FU.

Treatment of HCT116 cells with 5-FU after knockdown of *LINC01021* led to increased apoptosis compared to treatment with 5-FU and control siRNAs as determined by Annexin-V staining (Figure 5B). Furthermore, knockdown of *LINC01021* in HCT116 cells resulted in decreased proliferation after addition of two different concentrations of 5-FU as determined by realtime impedance measurement and subsequent cell counting (Figure 5C). In untreated cells *LINC01021* knockdown led to a minor decrease in cell proliferation, albeit without statistical significance (Figure 5C). Next, we analyzed whether loss of *LINC01021* also increases sensitivity to other chemotherapeutic agents. Therefore, we treated HCT116^*LINC01021KO*^ and HCT116^*LINC01021wt*^ clones with doxorubicin (DOXO) for 40 hours. Again, loss of *LINC01021* expression consistently led to an increase in Annexin-V/PI double-positive cells indicative of cells that had undergone apoptosis (Figure 6A, Supplementary Figure 4). Furthermore, colony formation assays with *LINC01021* wt and KO clones showed that cell viability was reduced upon treatment with DOXO for 2 to 10 hours followed by further cultivation for two days (Figure 6B). Furthermore, cellular impedance measurements showed that the sensitivity of *LINC01021* KO clones to doxorubicin was enhanced when compared to wt clones (Figure 6C). Taken together, these results indicate that loss of *LINC01021* RNA sensitizes cells to various chemotherapeutic treatments by rendering them more susceptible to apoptosis and/or permanent arrest. We did not detect effects of *LINC01021* deletion on p53 protein expression or induction of p21, which represents a marker for p53 transcriptional activity, after treatment with 5-FU (Figure 7A) or doxorubicin (Figure 7B). Therefore, *LINC01021* presumably does not act via modulating p53, as it was reported for other p53-induced lncRNAs, such as LINC-ROR [36].

Since loss or downregulation of *LINC01021* resulted in diminished cell survival and increased apoptosis of HCT116 cells after chemotherapeutic treatment, we asked whether ectopic expression of *LINC01021* would suppress apoptosis under these conditions. Therefore, we introduced expression constructs encoding two *LINC01021* transcript variants (isoforms #2 and #4, respectively, see Figure 2A) into the *p53*-proficient RKO and SW48 CRC cell lines, as well as their isogenic *p53* knockout derivatives, that, in contrast to HCT116 cells, are suitable host cell lines for the episomal pRTR vector [37] and allow tight regulation of *LINC01021* expression by doxycycline. Ectopic expression of *LINC01021* isoforms #2 and #4 in the *p53*-proficient SW48 CRC cell line did not result in altered p53 activation, nor altered induction of p21 upon treatment with 5-FU (Figure 7C). Ectopic expression of *LINC01021* did not significantly affect cell cycle distribution or apoptosis in RKO and SW48 cell lines treated with DOXO for 24 hours (Supplementary Figure 5A, 5B). Also induction of ectopic *LINC01021* itself did not result in obvious changes in cell cycle distribution.

The predominantly nuclear localization of all *LINC01021* isoforms (see Figure 4D) strongly suggested a nuclear function for *LINC01021* and prompted us to investigate whether *LINC01021* might be directly involved in the regulation of gene expression, e.g. through modulation of the p53-induced transcriptional program, as it has been reported for several other p53-induced lncRNAs (see introduction). We and others previously have shown that activation of p53 causes a mesenchymal-epithelial-transition (MET) in CRC cell lines, at least in part, by transcriptional activation of the miR-34 and miR-200 microRNA families and subsequent repression of EMT-inducing transcription factors, such as SNAIL and ZEB1 [38-41]. Therefore, we analyzed if loss of *LINC01021* affects p53-induced MET. However, we did not detect consistent differences in expression of epithelial and mesenchymal marker genes, such as E-Cadherin (*CDH1*) or Vimentin (*VIM*) between *LINC01021* wt and KO clones after treatment of cells with Nutlin-3 for 72 hours (Supplementary Figure 6A). Furthermore, ectopic expression of *LINC01021* alone or in combination with 5-FU treatment did not induce expression changes of epithelial or mesenchymal marker genes (data not shown).

Next, we analyzed whether gene repression after activation of p53 is affected by either loss or ectopic expression of *LINC01021*. Genes harboring E2F binding sites within their promoters, many of which are cell cycle regulatory genes involved in G_2_/M transition, are coordinately repressed upon activation of p53 by the p53-p21-DREAM-CDE/CHR pathway [42]. As some p53-induced lncRNAs, such as *linc-p21* and *PINT* have been implicated in gene repression by p53 [13, 43], we analyzed whether *LINC01021* affects repression of E2F target genes via the p53-p21-DREAM axis or by an independent mechanism. qPCR analyses after treatment with DOXO for 24 hours showed that repression of some E2F/DREAM target genes, such as *CIT*, was enhanced to a minor degree after loss of *LINC01021* (Supplementary Figure 6B). However, this effect did not consistently reach statistical significance. In addition, the repression of three other E2F/DREAM target genes (*MCM7, CDK2, CKS2*) was not affected significantly by loss of *LINC01021* (Supplementary Figure 6B). Also the ectopic expression of the *LINC01021* isoform #4 in RKO *p53*+/+ cells did not significantly alter expression of E2F/DREAM target genes either alone or in combination with 5-FU treatment (data not shown).

### *LINC01021* expression is associated with *p53* status in human CRC patient samples

**Figure 8:**
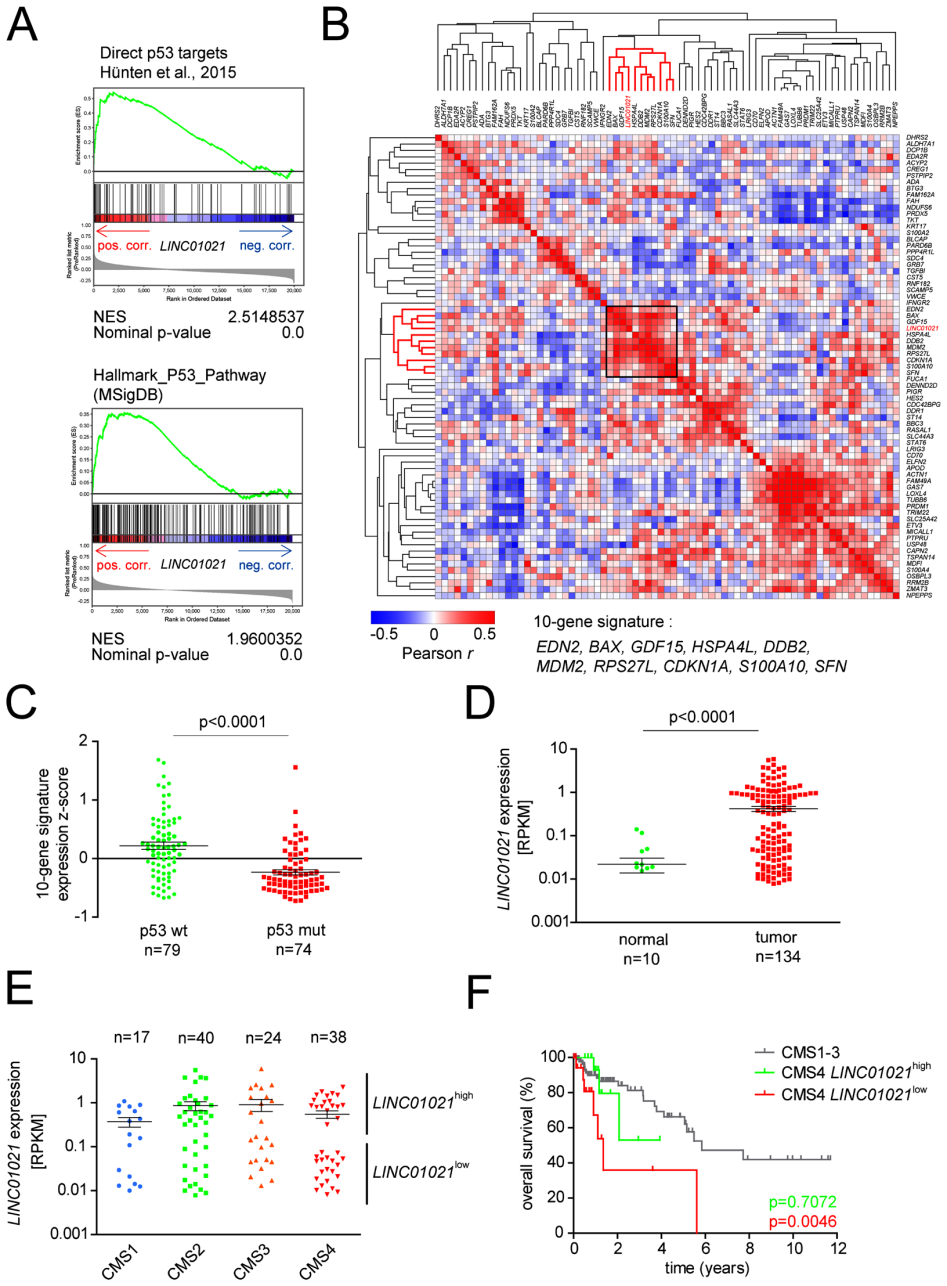
Assocation of *LINC01021* expression with wild-type p53 associated expression signatures and clinical outcome of CRC subtypes **(A)** Genes were preranked by expression correlation coefficient (Pearson *r*) with *LINC01021* in descending order from left (positive correlation) to right (negative correlation) based on publically available TCGA datasets from human colorectal tumors [59] and analyzed by GSEA. *LINC01021* expression values were obtained from [44]. Pos. corr.: positive correlation, neg. corr.: negative correlation. NES: normalized enrichment score. **(B)** Heatmap depicting the hierarchically clustered correlation matrix of pairwise expression correlation coefficients (Pearson *r*) between previously described direct p53 protein-coding target genes [30] and *LINC01021* based on TCGA COAD RNA expression data from 134 patient samples. **(C)** Averaged expression of ten p53 target genes selected in (B) based on normalized expression z-scores was calculated for each patient sample and associated with p53 mutational status. **(D)** *LINC01021* RNA expression levels within normal mucosa and primary tumors samples from CRC patients. Horizontal bars indicate mean +/- s.e.m. **(E)** Box plots showing *LINC01021* RNA expression levels in patient samples associated with the different CRC consensus molecular subtypes (CMS) [47]. Association of TCGA patient samples with CMS categories was obtained from the Colorectal Cancer Subtyping Consortium (CRCSC) at www.synapse.org. Horizontal bars indicate mean +/- s.e.m. **(F)** Kaplan-Meier plots showing overall survival of patients with primary CRCs classified as CMS1-3, or CMS4 with either high *LINC01021* or low *LINC01021* expression levels. P-values were calculated by log-rank (Mantel-Cox) test.

Since *LINC01021* expression was strongly associated with *p53* status in CRC cell lines, we determined whether *LINC01021* expression is associated with a distinct p53 status in CRC samples from patients. Therefore, we analyzed lncRNA expression data of colorectal tumor samples from 134 patients represented in the TCGA colorectal adenocarcinoma (COAD) patient cohort previously published by Yan et al. [44], and combined them with publically available TCGA COAD RNA-Seq data for protein-coding genes. Gene Set Enrichment Analyses (GSEA) showed a positive correlation between *LINC01021* and a set of direct p53 targets previously described by us [30], as well as the p53 Hallmark gene signature from the Molecular Signatures Database (MSigDB, [45, 46]), indicating that *LINC01021* expression is associated with p53 transcriptional activity in colorectal cancer (Figure 8A). Furthermore, GSEA showed a negative association between *LINC01021* expression in CRC samples and genes involved in processes known to be repressed by p53, such as G_2_/M transition, E2F/DREAM complex target genes [42], as well as genes involved in epithelial-mesenchymal transition [4] (Supplementary Figure 7).

Notably, *LINC01021* was co-expressed with a cluster of 10 direct p53 target genes that included well-studied p53 targets, such as *CDKN1A* (encoding p21), *MDM2*, *BAX* and *SFN/14-3-3σ* (Figure 8B). Since the information on *LINC01021* expression was not available for the large majority of patient samples with known *p53* mutational status, we used the averaged expression values of this 10-gene signature as a surrogate for *LINC01021* expression and determined whether the averaged expression of these 10 genes correlates with the p53 status of primary CRC samples represented within the TCGA COAD cohort (n=153). Thereby, we found that tumors with wild-type *p53* display significantly higher expression levels of the 10-gene signature and therefore presumably also have elevated *LINC01021* expression when compared to tumors with mutant *p53* (Figure 8C).

Next, we analyzed whether *LINC01021* expression in CRC samples associates with clinical parameters. Expression of *LINC01021* was significantly higher in tumor samples compared to normal mucosa (Figure 8D). Moreover, in tumor samples, *LINC01021* expression displayed a striking bimodal distribution, which was particularly pronounced in the mesenchymal CMS4 consensus molecular subtype [47] (Figure 8D, 8E). As previously reported [47], patients with tumors classified as CMS4 have a significantly poorer overall survival compared to patients with CMS subtype 1-3 tumors (data not shown). When we stratified CMS4 tumors into *LINC01021*^*high*^ or *LINC01021*^*low*^ CMS4 tumors, we found that patients with *LINC01021*^*low*^ CMS4 tumors had a significantly shorter overall survival than patients with CMS subtype 1-3, whereas patients with *LINC01021*^*high*^ CMS4 tumors did not have a significantly decreased survival compared to patients with CMS1-3 tumors (Figure 8F). Therefore, quantification of *LINC01021* expression may be relevant for the prognostication of patients with CMS4 tumors.

## DISCUSSION

We previously reported that the lncRNA *LINC01021* represents a direct p53 target gene [30]. Here, we show that *LINC01021* is consistently up-regulated in a p53-dependent manner in CRC cell lines upon treatment with p53-activating agents used for chemotherapy of CRC and other types of tumors. In addition, basal *LINC01021* expression was dependent on the presence of wild-type *p53* in CRC cell lines. Moreover, *LINC01021* expression is associated with a p53-dependent gene expression signature in CRC patient samples, further supporting the notion that *LINC01021* expression in CRC is dependent on the presence of wild-type *p53*. Using a CRISPR/Cas9 approach, we could show that deletions of promoter sequences eliminating the p53 binding site severely reduced both basal and p53-induced *LINC01021* expression in wild-type *p53* CRC cells. Furthermore, point mutations in the p53 binding site abrogated p53 responsiveness in luciferase reporter assays, implying that we identified the p53 binding motif for p53-dependent *LINC01021* expression. The deleted *LINC01021* promoter region harboring the p53 binding site is derived from a primate-specific repetitive DNA element belonging to the MER61C LTR subfamily of endogenous retrovirus 1 (ERV1). Furthermore, the *RP3-326I13/PINCR* lncRNA we had previously identified as a direct p53 target gene [30] and which recently has been reported to have a pro-survival function within the p53 network via regulation of a subset of p53 target genes involved in cell cycle arrest [48], contains a similar ERV1-derived MER61C LTR promoter element. These findings are in line with a bioinformatics analysis showing that ERV1 elements are enriched for putative p53 binding sites [29].

Notably, CRISPR/Cas9- and siRNA-mediated inactivation of *LINC01021* significantly enhanced sensitivity towards several different chemotherapeutic treatments. While this manuscript was in preparation, others reported that *LINC01021* (designated as *PURPL*) may act as a negative feedback regulator of basal p53 levels via HuR/MYBBP1A-mediated posttranslational regulation of p53 stability [49]. Here, we did not detect any effect of *LINC01021* inactivation or of its ectopic expression on basal and DNA damage-induced activity of p53 or p53 expression levels. The reasons for these discrepancies are currently unclear and may be resolved by further analyses. Nevertheless, both studies detected a markedly increased sensitivity of *LINC01021*-deficient CRC cells to DNA damaging agents used for chemotherapy in the clinics. We suggest that *LINC01021* acts as a downstream mediator of p53 function, without affecting p53 activity directly. *LINC01021* is presumably involved in integrating cellular repair and survival mechanisms that are activated after DNA damage occurs. The redistribution of ERV1-derived p53 response elements and the generation of novel lncRNA genes during primate evolution thus may have been co-opted as integral part of the cellular response to various forms of genotoxic stress. Given the substantial number of ERV1 LTRs with potentially functional p53 binding sites in the human genome, it will be interesting to determine if additional genes were rendered p53-responsive during evolution by insertion of ERV1 LTRs and how they contribute to p53-mediated tumor suppression.

As decreased levels of *LINC01021* enhance the response to chemotherapeutics quantification of *LINC01021* expression may have prognostic value. *LINC01021* expression was increased in CRC patient samples from the TCGA patient cohort compared to noncancerous tissue, yet highly variable and displayed a striking bimodal pattern that was particularly pronounced in the CMS4 subgroup. Interestingly, CMS4 tumors with low *LINC01021* expression were associated with poor patient survival. While the separation into *LINC01021*^high^ and *LINC01021*^low^ tumors may be attributable to p53 status, with low *LINC01021* levels reflecting mutational inactivation of p53, additional signaling pathways and mechanisms, for example epigenetic regulation or chromosomal aberrations may also influence *LINC01021* expression and thereby affect sensitivity to chemotherapeutic treatments. Further analyses are therefore warranted to illuminate the mechanisms of *LINC01021* function and regulation.

## MATERIALS AND METHODS

### Cell culture

The colorectal cancer cell lines HCT116, SW48 and RKO (all *p53+/+* or *p53-/-,* respectively) were obtained from Bert Vogelstein (Johns Hopkins Medical School, Baltimore). SW48 was cultured in DMEM medium with 10% FCS (Invitrogen), penicillin/streptomycin (10 units/ml), 5% CO_2_. HCT116 and RKO cells were cultured in McCoy´s medium with 10% FCS (Invitrogen), penicillin/streptomycin (10 units/ml), 5% CO_2_. Cells were treated with Nutlin-3 [10 μM], 5-FU [20 μg/ml or as indicated], doxorubicin [300 nM], etoposide [20 μM], or corresponding dilutions of DMSO. For conditional expression of p53 and *LINC01021* from pRTR vectors, doxycycline (Sigma) was used at a final concentration of 100 ng/ml.

### RNA isolation and qPCR analysis

Total RNA was prepared with the High Pure RNA Isolation Kit (Roche) according to the manufacturer´s protocol. cDNA was generated from 1 μg of total RNA per sample using anchored oligo(dT) primers (Verso cDNA Kit, Thermo Scientific). qPCR was performed by using the LightCycler 480 (Roche) and the Fast SYBR Green Master Mix (Applied Biosystems). Oligonucleotides used for qPCR are listed in Supplementary Table 1.

### Knockdown of *LINC01021*

For knock-down of *LINC01021*, siRNA pools consisting of 30 individual siRNAs (siPOOLS) that target all *LINC01021* splice variants were obtained from siTools (Martinsried, Germany). For RNA isolation after knockdown, HCT116 cells were seeded at 3x10^5^ cells per 6-well and transfected with 40 nM final siPOOL concentration. 24 hours after transfection, medium was supplemented with Nutlin-3 or 5-FU. RNA was harvested after 24 hours.

### CRISPR/Cas9-mediated inactivation of *LINC01021*

We designed two guide RNAs indicated in Supplementary Table 2 using the CRISPR design tool at tools.genome-engineering.org and cloned each of them via two complementary DNA oligonucleotides into the BbsI sites of pSp-Cas9-GFP to generate single-guide (sg) RNA expression plasmids, as described previously [50]. HCT116 cells were then transfected with 2.5 μg of each pSp-Cas9-sgRNA-GFP plasmid, or mock transfected with “empty” pSp-Cas9-GFP. 48 hours post transfection, GFP-positive cells were sorted into 96-wells using a FACSARIA cell sorter (BD Biosystems) and expanded as single-cell clones for two weeks. Mock transfected cells were treated in a similar manner to obtain *LINC01021* wild-type single-cell clones. Genomic DNA of individual clones was screened by PCR for appropriate deletions using primers indicated in Supplementary Table 3. PCR products were sequenced to verify the deletion of the p53 binding site. Clones with appropriate deletions within the *LINC01021* promoter were subsequently analyzed by qPCR to verify the loss of *LINC01021* expression.

### Quantification of apoptosis by Annexin-V/PI staining and FACS analysis

2x10^5^ cells from HCT116^LINC01021KO^ and HCT116^LINC01021wt^ clones were seeded in 12-well format. Doxorubicin was added after 48 hours. Apoptosis was analyzed after 40 hours with an Annexin-V staining kit (BD Biosciences) according to manufacturer´s instructions. Data from 10,000 cells was collected with an Accuri™ C6 flow cytometer (BD Biosciences). For measurement of apoptosis after knockdown of *LINC01021*, 1x10^5^ HCT116 *p53+/+* cells were seeded per well in 12-well format and transfected with siPOOLs at a final concentration of 40 nM. 24 hours after transfection, 5-FU was added to the medium. Apoptosis was analyzed after 24 hours.

### Subcellular fractionation

Subcellular fractionation into cytoplasmic and nuclear extracts was carried out as described previously [51]. Briefly, pelleted cells were lysed in hypotonic lysis buffer (10 mM Tris (pH 7.5), 10 mM NaCl, 3 mM MgCl_2_, 0.3% (vol/vol) NP-40 and 10% (vol/vol) glycerol) for 30 min on ice. An aliquot of the supernatant fraction was kept and the remaining nuclear pellet was washed three times with hypotonic lysis buffer. Subsequently, RNA of the supernatant fraction and the nuclear pellet fraction was isolated with the High Pure RNA Isolation Kit (Roche).

### Real-time impedance measurement

Determinations of cellular impedance as a measure of cell proliferation were performed with the Xcelligence system (Roche) as described previously [52]. HCT116 cells were seeded at 1x10^4^ cells per well of the E-plate, and treated with DMSO or doxorubicin after 24 hours. For impedance measurements after *LINC01021* knockdown, HCT116 cells were transfected with siPOOLs (40 nM final concentration) and seeded at 1x10^4^ cells per well of the E-plate. 24 hours after transfection, medium was supplemented with 5-FU at the indicated concentrations, or DMSO. To validate the results of the impedance measurement, the cells were simultaneously seeded in triplicates into 96-wells and the number of living cells was counted using trypan blue exclusion 48 hours after addition of 5-FU at the indicated concentrations, or DMSO.

### Luciferase reporter assay

A 412 bp fragment of the *LINC01021* promoter harboring the p53 binding site was PCR-amplified from genomic DNA of human diploid fibroblasts, ligated into the pBV-Luc vector and verified by sequencing. Mutagenesis of the p53 binding site was carried out with the QuickChange mutagenesis kit according to manufacturer´s instructions. Oligonucleotides used for cloning and mutagenesis are listed in Supplementary Table 3. Luciferase assays were performed 48 hours after transfection using the Dual Luciferase Reporter assay system (Promega) according to the manufacturer´s protocol. Fluorescence intensities were measured with an Orion II luminometer (Berthold) in a 96-well format and analyzed with the SIMPLICITY software package (DLR).

### Western blot analysis

Cells were lysed in RIPA lysis buffer (50 mM Tris/HCl, pH 8.0, 250 mM NaCl, 1% NP40, 0.5% sodium deoxycholate, 0.1% sodium dodecylsulfate, complete mini protease inhibitor tablets (Roche)). Lysates were sonicated and centrifuged at 16,060 g for 15 min at 4°C. 20 μg of whole cell lysate per lane were separated using 10% SDS-acrylamide gels and transferred on Immobilon PVDF membranes (Millipore). Antibodies used were specific for p53 (DO-1), p21 (BD Pharmingen) and β-actin (Sigma, A2066).

### Colony formation assays

Cells were seeded at 2x10^5^ cells per 6-well. After 24 hours, doxorubicin was added for the indicated periods. Cells were washed once with HBSS, new medium was added and cells were allowed to recover for two days before fixation and crystal violet staining

### Cloning and conditional expression of *LINC01021* isoforms

*LINC01021* splice variants were PCR amplified using oligo-dT-primed cDNAs from SW480 cells after conditional expression of p53 [30] using primers listed in Supplementary Table 3 and cloned into episomal pRTR vectors described previously [37, 40]. All plasmids were verified by sequencing. Polyclonal cell pools of SW48 *p53+/+* and RKO *p53+/+* cells, as well as their isogenic p53 KO derivatives, for conditional expression were generated by transfection of pRTR vectors using Fugene6 (Roche) and selection in 2 μg/ml puromycin (Sigma) for 10 days.

### Cell cycle analysis by propidium iodide (PI) staining

2x10^5^ cells from HCT116^*LINC01021KO*^ and HCT116^*LINC01021wt*^ clones were seeded in 12-well format. 5-FU [20 μg/ml] was added after 48 hours for 24 hours. For PI staining of SW48 or RKO cells ectopically expressing *LINC01021*, doxycycline (DOX) was added 12 hours after seeding. After 32 hours (pre-treatment), doxorubicin (DOXO) was added for the indicated time periods. Both the supernatant and attached cell fractions were collected and combined after trypsination. Cells were washed once in HBSS and fixed in ice-cold ethanol (70%) overnight at -20°C. Fixed cells were washed with PBS and resuspended in PI staining solution. Cell cycle distribution of the cells was measured using an Accuri™ C6 flow cytometry instrument (BD Biosciences) and analyzed with the CFlow® software.

### *In silico* analysis of human colorectal cancer samples

RNA expression data from colorectal adenocarcinoma (COAD) patient samples were obtained from the TCGA data portal at https://cancergenome.nih.gov/. RNA-Seq by Expectation-Maximization (RSEM) normalized expression values from the Illumina RNASeqV2 (genes) datasets were used. LncRNA expression data from the TCGA COAD cohort were previously published in [44] and kindly provided by Lin Zhang (Center for Research on Reproduction & Women’s Health, Perelman School of Medicine, University of Pennsylvania). 134 TCGA COAD samples with detectable *LINC01021* expression were included in the analyses. Association of TCGA patient samples with the different CMS categories was obtained from the Colorectal Cancer Subtyping Consortium (CRCSC) at www.synapse.org. Information on *p53* mutation status of patient samples of the TCGA COAD cohort was obtained from http://www.cbioportal.org/. Gene Set Enrichment Analysis (GSEA) was performed using the GSEA software [45]. Hallmark gene sets for GSEA were obtained from the Molecular Signatures Database (Broad Institute) [46]. The hierarchically clustered heatmap of expression correlations was generated with GENE-E (Broad Institute).

### Bioinformatics analyses of NGS data

ChIP-Seq and RNA-Seq data obtained after ectopic expression of p53 in SW480 cells and previously published [30] were re-analyzed by mapping to the hg38 human genome assembly with the CLC Genomics Workbench software (QIAGEN) using default settings. The generated BAM files were further analyzed using the software packages implemented on the Galaxy bioinformatics server at usegalaxy.org. ChIP-Seq peak calling was performed with the MACS2 algorithm [53, 54] using default settings. Generation of heatmaps depicting p53 binding events in selected genomic regions was performed using the Deeptools software packages [55] implemented at Galaxy. DNA binding motif analysis was performed using MEME [32]. Locations of repetitive DNA elements at the *LINC01021* and *RP3-326I.13* genomic loci were identified using the Repeatmasker software (http://www.repeatmasker.org/) and the Dfam database [33].

### Statistical analysis

A Student´s *t*-test (unpaired, two-tailed) was used to determine significant differences between two groups of samples (applied for qPCR, cell cycle and cell counting analyses, luciferase reporter assays and for differential gene expression analyses using TCGA). For correlation analyses, a Pearson´s correlation was applied. P-values < 0.05 were considered as significant (^*^: p < 0.05; ^**^: p < 0.01; ^***^: p < 0.001).

## SUPPLEMENTARY MATERIALS FIGURES AND TABLES









## Abbreviations

5-FU: 5-fluorouracil
cDNA: complementary DNA
ChIP: Chromatin immunoprecipitation
CMS: Consensus Molecular Subtype
COAD: Colorectal adenocarcinoma
CRC: Colorectal cancer
CRISPR: Clustered Regularly Interspaced Short Palindromic Repeats
DOX: Doxycycline
DOXO: Doxorubicin
EMT: Epithelial-mesenchymal transition
ERV: Endogenous retrovirus
GSEA: Gene set enrichment analysis
Kbp: kilobasepair
Lnc: Long non-coding
LTR: Long terminal repeat
MACS: Model-based Analysis of ChIP-Seq data
MEME: Multiple Em for Motif Elicitation
MER: Medium Reiteration Frequency Interspersed Repeat
MET: Mesenchymal-epithelial transition
NGS: Next generation sequencing
nt: nucleotides
RPKM: Reads per kilobase per million reads
RSEM: RNA-Seq by Expectation-Maximization
sgRNA: Single-guide RNA
siRNA: Small interfering RNA
TCGA: The Cancer Genome Atlas
TE: Transposable element
TSS: Transcription start site

## References

[1] Soussi T (2011). TP53 mutations in human cancer: database reassessment and prospects for the next decade. Adv Cancer Res.

[2] Cheok CF, Verma CS, Baselga J, Lane DP (2011). Translating p53 into the clinic. Nat Rev Clin Oncol.

[3] Riley T, Sontag E, Chen P, Levine A (2008). Transcriptional control of human p53-regulated genes. Nat Rev Mol Cell Biol.

[4] Hermeking H (2012). MicroRNAs in the p53 network: micromanagement of tumour suppression. Nat Rev Cancer.

[5] Zhang A, Xu M, Mo YY (2014). Role of the lncRNA-p53 regulatory network in cancer. J Mol Cell Biol.

[6] Grossi E, Sanchez Y, Huarte M (2016). Expanding the p53 regulatory network: LncRNAs take up the challenge. Biochim Biophys Acta.

[7] Tay Y, Rinn J, Pandolfi PP (2014). The multilayered complexity of ceRNA crosstalk and competition. Nature.

[8] Khalil AM, Guttman M, Huarte M, Garber M, Raj A, Rivea Morales D, Thomas K, Presser A, Bernstein BE, van Oudenaarden A, Regev A, Lander ES, Rinn JL (2009). Many human large intergenic noncoding RNAs associate with chromatin-modifying complexes and affect gene expression. Proc Natl Acad Sci U S A.

[9] Ulitsky I, Bartel DP (2013). lincRNAs: genomics, evolution, and mechanisms. Cell.

[10] Yoon JH, Abdelmohsen K, Gorospe M (2013). Posttranscriptional gene regulation by long noncoding RNA. J Mol Biol.

[11] Guttman M, Amit I, Garber M, French C, Lin MF, Feldser D, Huarte M, Zuk O, Carey BW, Cassady JP, Cabili MN, Jaenisch R, Mikkelsen TS (2009). Chromatin signature reveals over a thousand highly conserved large non-coding RNAs in mammals. Nature.

[12] Hung T, Wang Y, Lin MF, Koegel AK, Kotake Y, Grant GD, Horlings HM, Shah N, Umbricht C, Wang P, Wang Y, Kong B, Langerod A (2011). Extensive and coordinated transcription of noncoding RNAs within cell-cycle promoters. Nat Genet.

[13] Huarte M, Guttman M, Feldser D, Garber M, Koziol MJ, Kenzelmann-Broz D, Khalil AM, Zuk O, Amit I, Rabani M, Attardi LD, Regev A, Lander ES (2010). A large intergenic noncoding RNA induced by p53 mediates global gene repression in the p53 response. Cell.

[14] Leveille N, Melo CA, Rooijers K, Diaz-Lagares A, Melo SA, Korkmaz G, Lopes R, Akbari Moqadam F, Maia AR, Wijchers PJ, Geeven G, den Boer ML, Kalluri R (2015). Genome-wide profiling of p53-regulated enhancer RNAs uncovers a subset of enhancers controlled by a lncRNA. Nat Commun.

[15] Sanchez Y, Segura V, Marin-Bejar O, Athie A, Marchese FP, Gonzalez J, Bujanda L, Guo S, Matheu A, Huarte M (2014). Genome-wide analysis of the human p53 transcriptional network unveils a lncRNA tumour suppressor signature. Nat Commun.

[16] Younger ST, Kenzelmann-Broz D, Jung H, Attardi LD, Rinn JL (2015). Integrative genomic analysis reveals widespread enhancer regulation by p53 in response to DNA damage. Nucleic Acids Res.

[17] Yoon JH, Abdelmohsen K, Srikantan S, Yang X, Martindale JL, De S, Huarte M, Zhan M, Becker KG, Gorospe M (2012). LincRNA-p21 suppresses target mRNA translation. Mol Cell.

[18] Schmitt AM, Garcia JT, Hung T, Flynn RA, Shen Y, Qu K, Payumo AY, Peres-da-Silva A, Broz DK, Baum R, Guo S, Chen JK, Attardi LD, Chang HY (2016). An inducible long noncoding RNA amplifies DNA damage signaling. Nat Genet.

[19] Adriaens C, Standaert L, Barra J, Latil M, Verfaillie A, Kalev P, Boeckx B, Wijnhoven PW, Radaelli E, Vermi W, Leucci E, Lapouge G, Beck B (2016). p53 induces formation of NEAT1 lncRNA-containing paraspeckles that modulate replication stress response and chemosensitivity. Nat Med.

[20] Faulkner GJ, Kimura Y, Daub CO, Wani S, Plessy C, Irvine KM, Schroder K, Cloonan N, Steptoe AL, Lassmann T, Waki K, Hornig N, Arakawa T (2009). The regulated retrotransposon transcriptome of mammalian cells. Nat Genet.

[21] Jacques PE, Jeyakani J, Bourque G (2013). The majority of primate-specific regulatory sequences are derived from transposable elements. PLoS Genet.

[22] Kapusta A, Kronenberg Z, Lynch VJ, Zhuo X, Ramsay L, Bourque G, Yandell M, Feschotte C (2013). Transposable elements are major contributors to the origin, diversification, and regulation of vertebrate long noncoding RNAs. PLoS Genet.

[23] Kelley D, Rinn J (2012). Transposable elements reveal a stem cell-specific class of long noncoding RNAs. Genome Biol.

[24] Flockhart RJ, Webster DE, Qu K, Mascarenhas N, Kovalski J, Kretz M, Khavari PA (2012). BRAFV600E remodels the melanocyte transcriptome and induces BANCR to regulate melanoma cell migration. Genome Res.

[25] Gibb EA, Warren RL, Wilson GW, Brown SD, Robertson GA, Morin GB, Holt RA (2015). Activation of an endogenous retrovirus-associated long non-coding RNA in human adenocarcinoma. Genome Med.

[26] Wylie A, Jones AE, D’Brot A, Lu WJ, Kurtz P, Moran JV, Rakheja D, Chen KS, Hammer RE, Comerford SA, Amatruda JF, Abrams JM (2016). p53 genes function to restrain mobile elements. Genes Dev.

[27] Botcheva K, McCorkle SR (2014). Cell context dependent p53 genome-wide binding patterns and enrichment at repeats. PloS One.

[28] Leonova KI, Brodsky L, Lipchick B, Pal M, Novototskaya L, Chenchik AA, Sen GC, Komarova EA, Gudkov AV (2013). p53 cooperates with DNA methylation and a suicidal interferon response to maintain epigenetic silencing of repeats and noncoding RNAs. Proc Natl Acad Sci U S A.

[29] Wang T, Zeng J, Lowe CB, Sellers RG, Salama SR, Yang M, Burgess SM, Brachmann RK, Haussler D (2007). Species-specific endogenous retroviruses shape the transcriptional network of the human tumor suppressor protein p53. Proc Natl Acad Sci U S A.

[30] Hünten S, Kaller M, Drepper F, Oeljeklaus S, Bonfert T, Erhard F, Dueck A, Eichner N, Friedel CC, Meister G, Zimmer R, Warscheid B, Hermeking H (2015). p53-Regulated Networks of Protein, mRNA, miRNA, and lncRNA Expression Revealed by Integrated Pulsed Stable Isotope Labeling With Amino Acids in Cell Culture (pSILAC) and Next Generation Sequencing (NGS) Analyses. Mol Cell Proteomics.

[31] Harris CR, Dewan A, Zupnick A, Normart R, Gabriel A, Prives C, Levine AJ, Hoh J (2009). p53 responsive elements in human retrotransposons. Oncogene.

[32] Bailey TL (2002). Discovering novel sequence motifs with MEME. Curr Protoc Bioinformatics.

[33] Hubley R, Finn RD, Clements J, Eddy SR, Jones TA, Bao W, Smit AF, Wheeler TJ (2016). The Dfam database of repetitive DNA families. Nucleic Acids Res.

[34] Quinn JJ, Chang HY (2016). Unique features of long non-coding RNA biogenesis and function. Nat Rev Genet.

[35] Schlackow M, Nojima T, Gomes T, Dhir A, Carmo-Fonseca M, Proudfoot NJ (2017). Distinctive Patterns of Transcription and RNA Processing for Human lincRNAs. Mol Cell.

[36] Zhang A, Zhou N, Huang J, Liu Q, Fukuda K, Ma D, Lu Z, Bai C, Watabe K, Mo YY (2013). The human long non-coding RNA-RoR is a p53 repressor in response to DNA damage. Cell Res.

[37] Jackstadt R, Roh S, Neumann J, Jung P, Hoffmann R, Horst D, Berens C, Bornkamm GW, Kirchner T, Menssen A, Hermeking H (2013). AP4 is a mediator of epithelial-mesenchymal transition and metastasis in colorectal cancer. J Exp Med.

[38] Burk U, Schubert J, Wellner U, Schmalhofer O, Vincan E, Spaderna S, Brabletz T (2008). A reciprocal repression between ZEB1 and members of the miR-200 family promotes EMT and invasion in cancer cells. EMBO Rep.

[39] Wellner U, Schubert J, Burk UC, Schmalhofer O, Zhu F, Sonntag A, Waldvogel B, Vannier C, Darling D, zur Hausen A, Brunton VG, Morton J, Sansom O (2009). The EMT-activator ZEB1 promotes tumorigenicity by repressing stemness-inhibiting microRNAs. Nat Cell Biol.

[40] Siemens H, Jackstadt R, Hunten S, Kaller M, Menssen A, Gotz U, Hermeking H (2011). miR-34 and SNAIL form a double-negative feedback loop to regulate epithelial-mesenchymal transitions. Cell Cycle.

[41] Singh A, Settleman J (2010). EMT, cancer stem cells and drug resistance: an emerging axis of evil in the war on cancer. Oncogene.

[42] Fischer M, Quaas M, Steiner L, Engeland K (2016). The p53-p21-DREAM-CDE/CHR pathway regulates G2/M cell cycle genes. Nucleic Acids Res.

[43] Marin-Bejar O, Marchese FP, Athie A, Sanchez Y, Gonzalez J, Segura V, Huang L, Moreno I, Navarro A, Monzo M, Garcia-Foncillas J, Rinn JL, Guo S, Huarte M (2013). Pint lincRNA connects the p53 pathway with epigenetic silencing by the Polycomb repressive complex 2. Genome Biol.

[44] Yan X, Hu Z, Feng Y, Hu X, Yuan J, Zhao SD, Zhang Y, Yang L, Shan W, He Q, Fan L, Kandalaft LE, Tanyi JL (2015). Comprehensive Genomic Characterization of Long Non-coding RNAs across Human Cancers. Cancer Cell.

[45] Subramanian A, Tamayo P, Mootha VK, Mukherjee S, Ebert BL, Gillette MA, Paulovich A, Pomeroy SL, Golub TR, Lander ES, Mesirov JP (2005). Gene set enrichment analysis: a knowledge-based approach for interpreting genome-wide expression profiles. Proc Natl Acad Sci U S A.

[46] Liberzon A, Birger C, Thorvaldsdottir H, Ghandi M, Mesirov JP, Tamayo P (2015). The Molecular Signatures Database (MSigDB) hallmark gene set collection. Cell Syst.

[47] Guinney J, Dienstmann R, Wang X, de Reynies A, Schlicker A, Soneson C, Marisa L, Roepman P, Nyamundanda G, Angelino P, Bot BM, Morris JS, Simon IM (2015). The consensus molecular subtypes of colorectal cancer. Nat Med.

[48] Chaudhary R, Gryder B, Woods WS, Subramanian M, Jones MF, Li XL, Jenkins LM, Shabalina SA, Mo M, Dasso M, Yang Y, Wakefield LM, Zhu Y (2017). Prosurvival long noncoding RNA PINCR regulates a subset of p53 targets in human colorectal cancer cells by binding to Matrin 3. Elife.

[49] Li XL, Subramanian M, Jones MF, Chaudhary R, Singh DK, Zong X, Gryder B, Sindri S, Mo M, Schetter A, Wen X, Parvathaneni S, Kazandjian D (2017). Long Noncoding RNA PURPL Suppresses Basal p53 Levels and Promotes Tumorigenicity in Colorectal Cancer. Cell Rep.

[50] Ran FA, Hsu PD, Wright J, Agarwala V, Scott DA, Zhang F (2013). Genome engineering using the CRISPR-Cas9 system. Nat Protoc.

[51] Gagnon KT, Li L, Janowski BA, Corey DR (2014). Analysis of nuclear RNA interference in human cells by subcellular fractionation and Argonaute loading. Nat Protoc.

[52] Siemens H, Jackstadt R, Kaller M, Hermeking H (2013). Repression of c-Kit by p53 is mediated by miR-34 and is associated with reduced chemoresistance, migration and stemness. Oncotarget.

[53] Feng J, Liu T, Qin B, Zhang Y, Liu XS (2012). Identifying ChIP-seq enrichment using MACS. Nat Protoc.

[54] Zhang Y, Liu T, Meyer CA, Eeckhoute J, Johnson DS, Bernstein BE, Nusbaum C, Myers RM, Brown M, Li W, Liu XS (2008). Model-based analysis of ChIP-Seq (MACS). Genome Biol.

[55] Ramirez F, Ryan DP, Gruning B, Bhardwaj V, Kilpert F, Richter AS, Heyne S, Dundar F, Manke T (2016). deepTools2: a next generation web server for deep-sequencing data analysis. Nucleic Acids Res.

[56] Diaz-Lagares A, Crujeiras AB, Lopez-Serra P, Soler M, Setien F, Goyal A, Sandoval J, Hashimoto Y, Martinez-Cardus A, Gomez A, Heyn H, Moutinho C, Espada J (2016). Epigenetic inactivation of the p53-induced long noncoding RNA TP53 target 1 in human cancer. Proc Natl Acad Sci U S A.

[57] Wang L, Bu P, Ai Y, Srinivasan T, Chen HJ, Xiang K, Lipkin SM, Shen X (2016). A long non-coding RNA targets microRNA miR-34a to regulate colon cancer stem cell asymmetric division. Elife.

[58] Li W, Cowley A, Uludag M, Gur T, McWilliam H, Squizzato S, Park YM, Buso N, Lopez R (2015). The EMBL-EBI bioinformatics web and programmatic tools framework. Nucleic Acids Res.

[59] The TCGA Network (2012). Comprehensive molecular characterization of human colon and rectal cancer. Nature.

