# Supplementary material for: Loss of p53-inducible long non-coding RNA LINC01021 increases chemosensitivity
Source: Oncotarget. 2017 Nov 1;8(61):102783–800. doi: 10.18632/oncotarget.22245 (PMC5732690; doi:10.18632/oncotarget.22245)
Supplement: Supplementary file 1 [file oncotarget-08-102783-s001.pdf]

Loss of p53-inducible long non-coding RNA *LINC01021* increases chemosensitivity

SUPPLEMENTARY MATERIALS

REFERENCES

1. Hünten S, Kaller M, Drepper F, Oeljeklaus S, Bonfert T, Erhard F, Dueck A, Eichner N, Friedel CC, Meister G, Zimmer R, Warscheid B, Hermeking H. p53-Regulated Networks of Protein, mRNA, miRNA, and lncRNA Expression Revealed by Integrated Pulsed Stable Isotope Labeling With Amino Acids in Cell Culture (pSILAC) and Next Generation Sequencing (NGS) Analyses. Mol Cell Proteomics. 2015; 14:2609-29.
2. Hubley R, Finn RD, Clements J, Eddy SR, Jones TA, Bao W, Smit AF, Wheeler TJ. The Dfam database of repetitive DNA families. Nucleic Acids Res. 2016; 44:D81-9.
3. Li W, Cowley A, Uludag M, Gur T, McWilliam H, Squizzato S, Park YM, Buso N, Lopez R. The EMBL-EBI bioinformatics web and programmatic tools framework. Nucleic Acids Res. 2015; 43:W580-4.
4. The TCGA Network. Comprehensive molecular characterization of human colon and rectal cancer. Nature. 2012; 487:330-7.
5. Yan X, Hu Z, Feng Y, Hu X, Yuan J, Zhao SD, Zhang Y, Yang L, Shan W, He Q, Fan L, Kandalaft LE, Tanyi JL, et al. Comprehensive Genomic Characterization of Long Non-coding RNAs across Human Cancers. Cancer Cell. 2015; 28:529-40.
6. Liberzon A, Birger C, Thorvaldsdottir H, Ghandi M, Mesirov JP, Tamayo P. The Molecular Signatures Database (MSigDB) hallmark gene set collection. Cell Syst. 2015; 1:417-25.
7. Fischer M, Quaas M, Steiner L, Engeland K. The p53-p21-DREAM-CDE/CHR pathway regulates G2/M cell cycle genes. Nucleic Acids Res. 2016; 44:164-74.

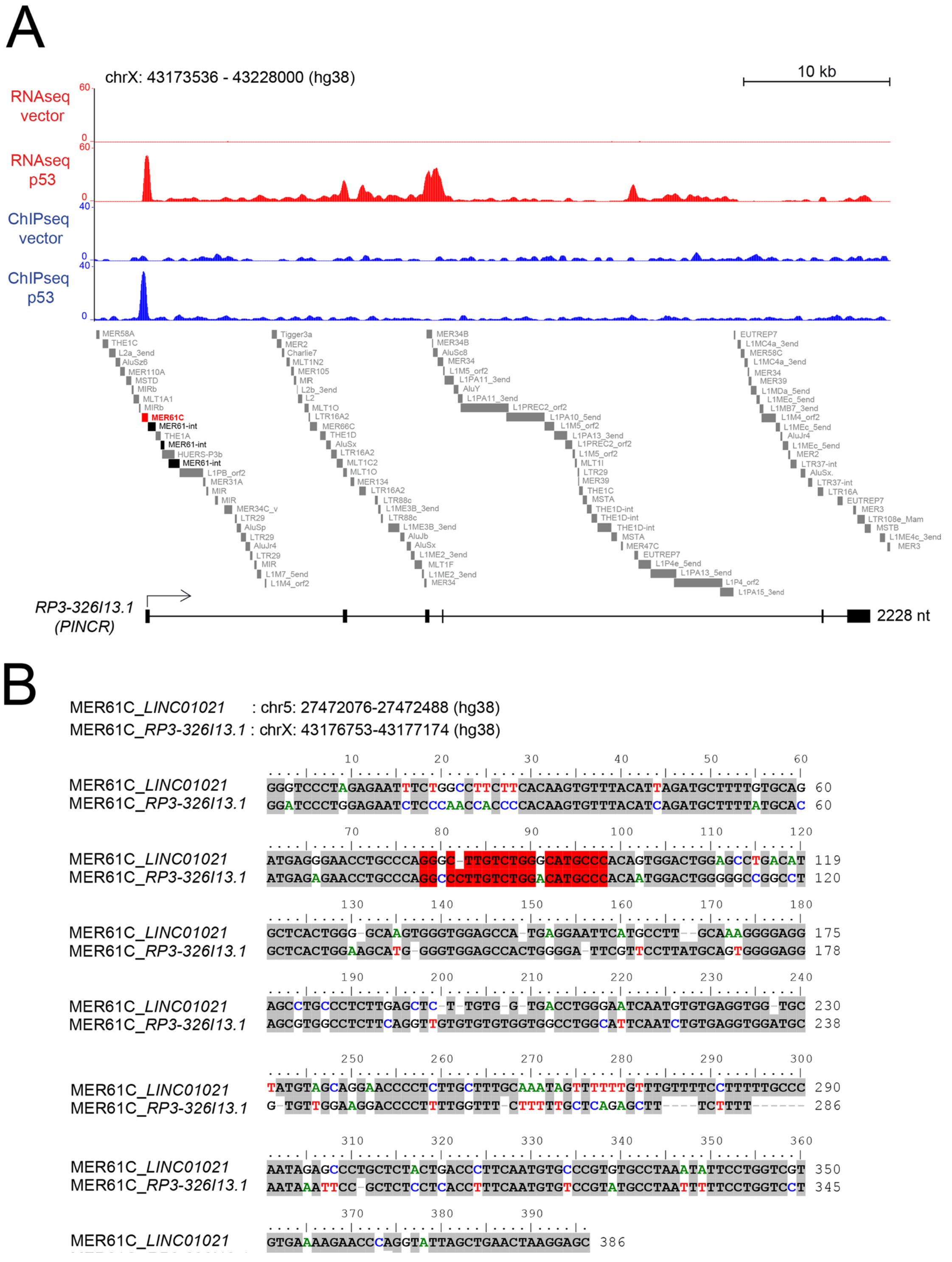

Supplementary Figure 1: The *RP3-326I13.1/PINCR* and *LINC01021* promoter regions bound by p53 are composed of highly similar ERV1-derived MER61C LTR elements. (A) RNA-Seq and ChIP-Seq results obtained after ectopic expression of p53 in SW480 cells were mapped to the *RP3-326I13.1/PINCR* genomic locus. Y-axes indicate read-numbers. NGS-data were generated and published previously [1]. Genomic coordinates of repetitive DNA elements were obtained using the Dfam database [2]. The MER61C element harboring the p53 binding site associated with the *RP3-326I13.1/PINCR* promoter is indicated in red. Additional MER61 DNA sequences are shown in black, other repetitive elements in grey. **(B)** Alignment of the MER61C sequences from the *LINC01021* and *RP3-326I13.1/PINCR* promoters. The p53 binding site is highlighted in red. Pairwise sequence alignment was performed with Promoterwise [3] and edited with the BioEdit software.

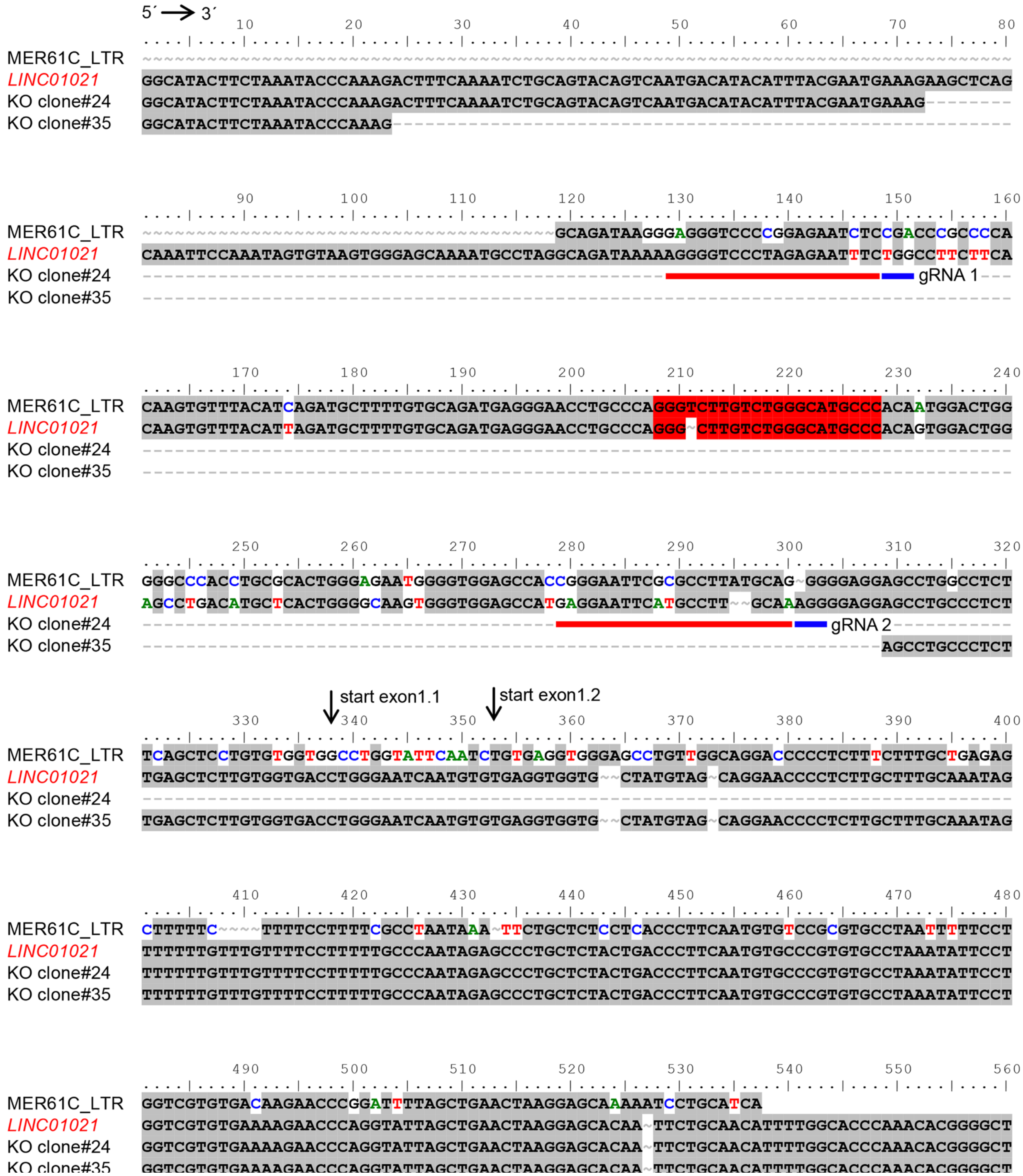

Supplementary Figure 2: CRISPR/Cas9-mediated deletion within the *LINC01021* promoter region. Alignment of the *LINC01021* promoter sequence, the MER61C profile hidden Markov model (HMM) obtained from the Dfam database and DNA sequences obtained from the two indicated single-cell HCT116 clones. The positions of the two guide RNAs (gRNAs) and the adjacent PAM sequence are indicated below the *LINC01021* sequence as red and blue horizontal bars, respectively. Guide RNAs were designed to target sequences significantly divergent (i.e. at least 5 mismatches) between MER61C HMM and *LINC01021* promoter, and/or lacking an adjacent PAM in the MER61C HMM, in order to minimize potential targeting of other MER61C repeats. The p53 binding sites within the *LINC01021* promoter and the MER61C HMM are high-lighted in red. The two ENSEMBL-annotated alternative transcriptional start sites of exon 1 of the *LINC01021* isoforms 1-5 are indicated with vertical arrows.

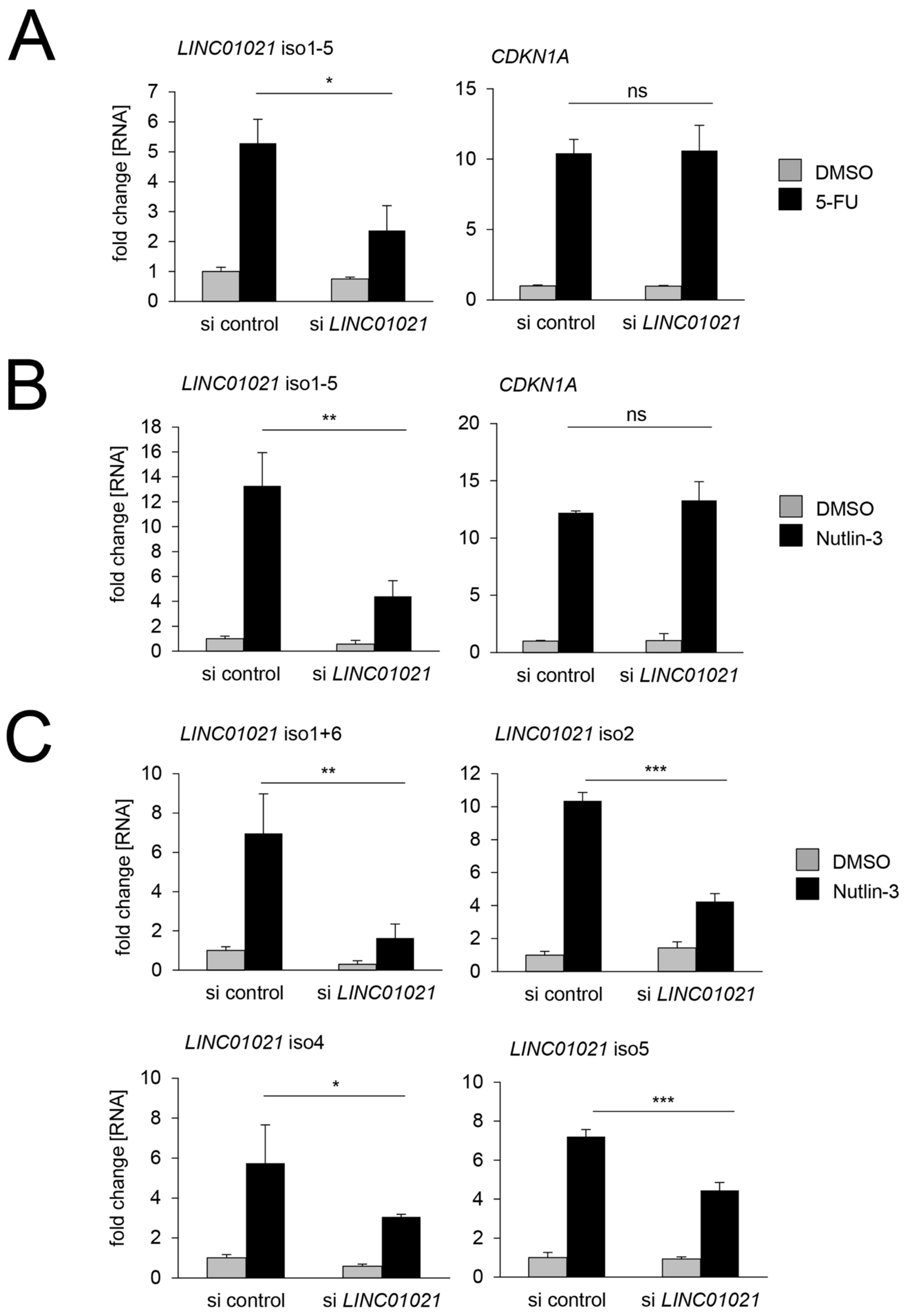

Supplementary Figure 3: Knockdown of *LINC01021* isoform expression using siRNA pools. HCT116 cells were transfected with siRNA pools at 40 nM final concentration. 24 hours after transfection, medium was supplemented with **(A)** 5-FU, **(B)** Nutlin-3, or DMSO. RNA was harvested after 24 hours. *LINC01021* and *CDKN1A* expression was normalized to expression in DMSO-treated cells transfected with control siRNAs and expression of *GAPDH*. **(C)** Expression of the indicated *LINC01021* splice variants was analyzed as in (A and B). Results represent the mean +/- s.d. (n =3).

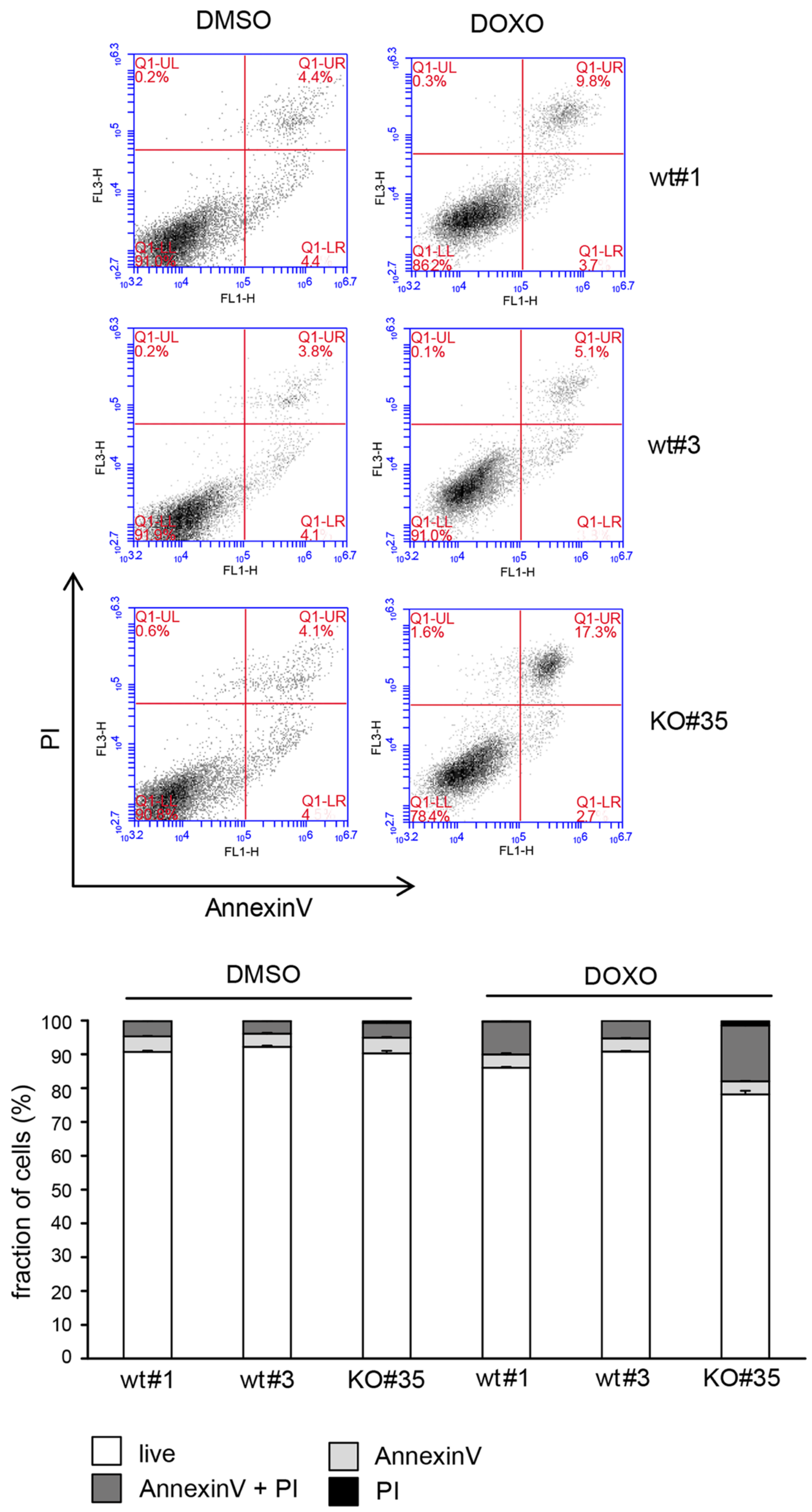

Supplementary Figure 4: Loss of *LINC01021* sensitizes HCT116 cells to doxorubicin. Apoptosis was measured by FACS analysis after AnnexinV/PI staining after treatment of *LINC01021* wt and KO clones with DMSO (control) or doxorubicin (DOXO) for 24 hours. A representative FACS plot for each clone and condition is shown. The quantification of biological replicates *(n=3)* is shown below.

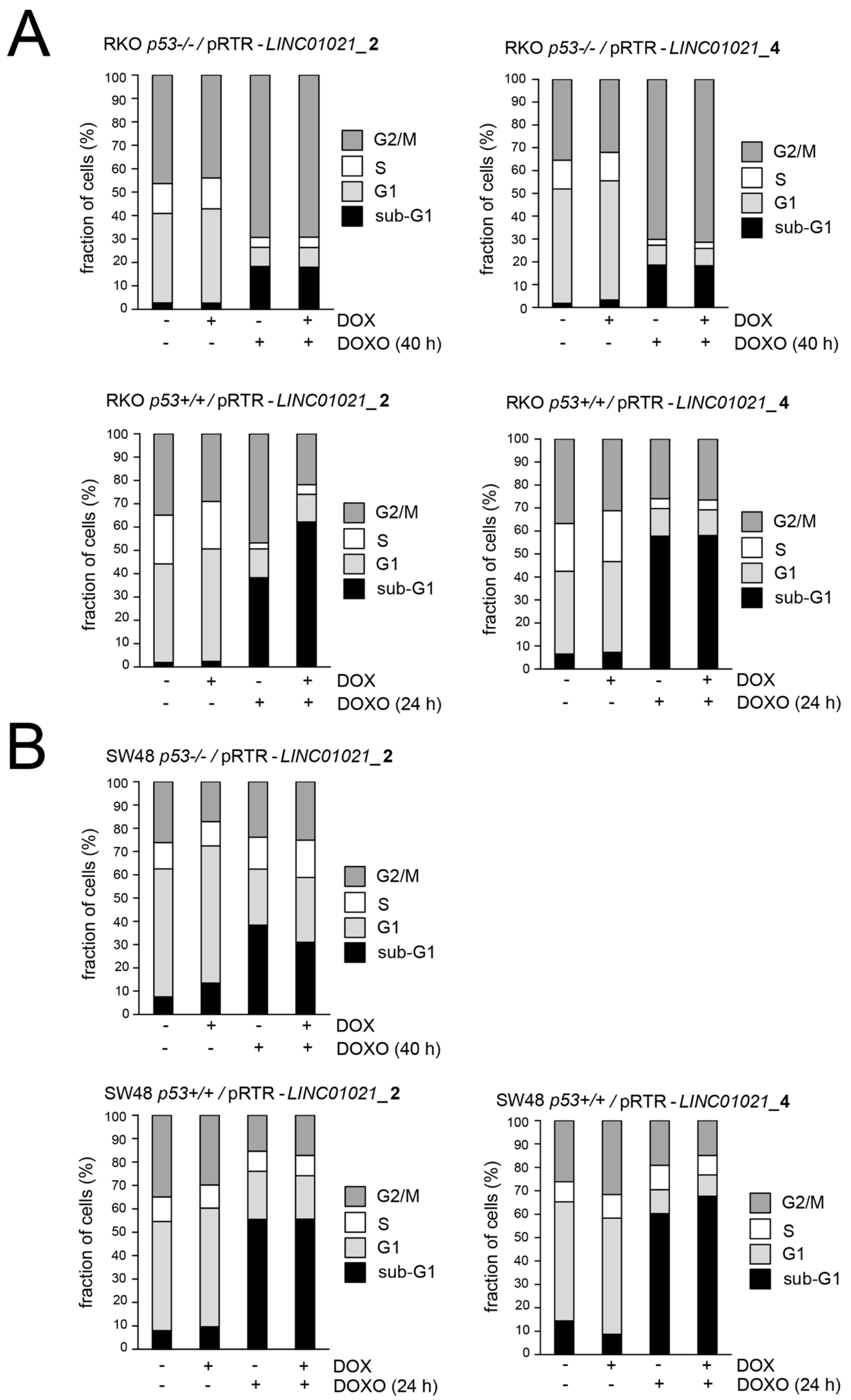

Supplementary Figure 5: Effect of *LINC01021* isoforms on doxorubicin-mediated cell cycle alterations. DNA content analysis by FACS of **(A)** RKO *p53-/-* or *p53+/+* and **(B)** SW48 *p53-/-* or *p53+/+* CRC cell lines ectopically expressing the indicated *LINC0121* isoforms after treatment with DMSO or doxorubicin (DOXO) for the indicated time periods. *LINC01021* expression was induced by addition of doxycycline (DOX) for 32 hours before addition of DOXO.

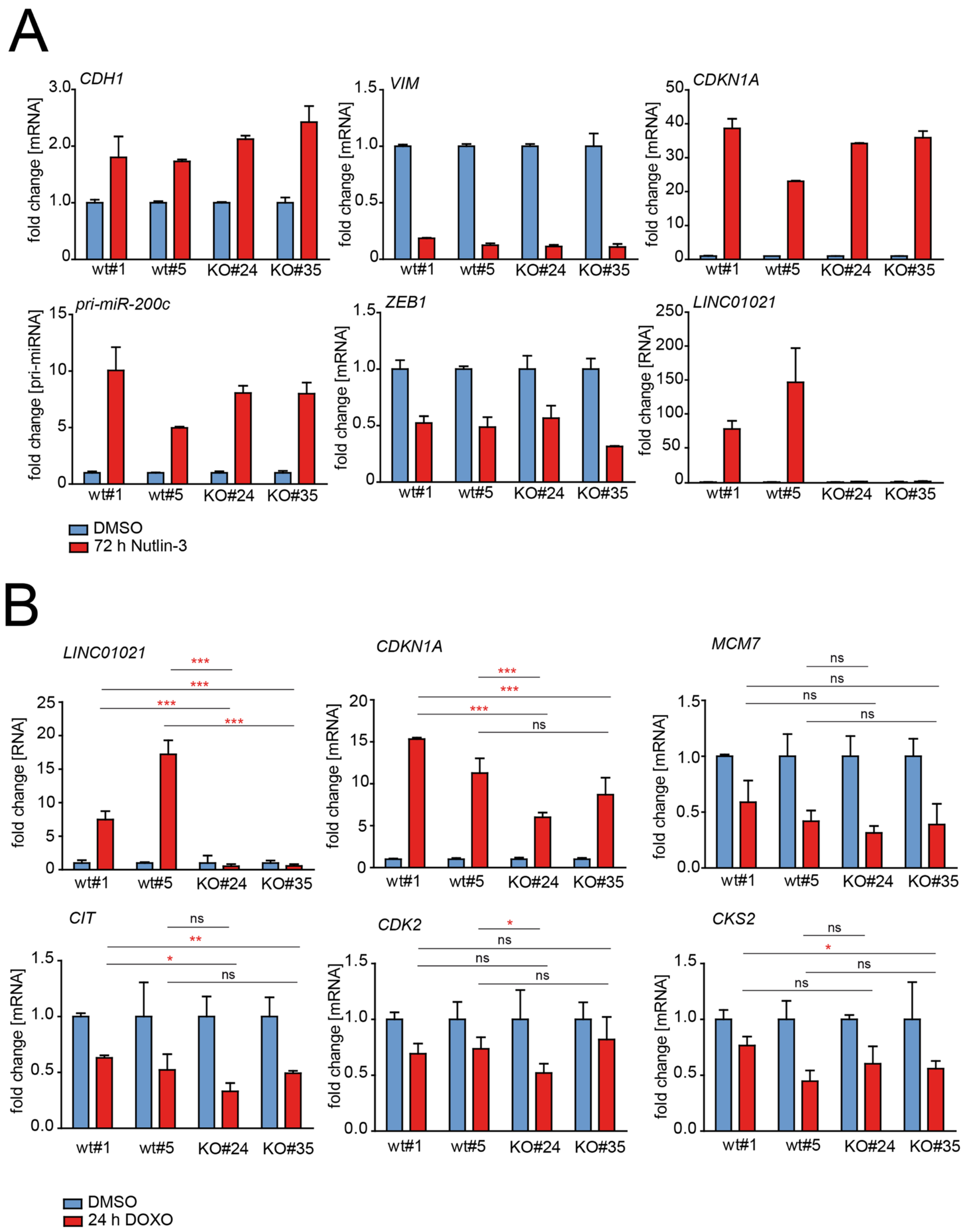

Supplementary Figure 6: Analysis of the influence of *LINC01021* on p53-mediated differential expression of EMT or G_2/M transition related genes. Expression of the indicated mRNAs, pri-miRNAs and lncRNAs was determined by qPCR analyses after treatment of *LINC01021* wt and KO clones with **(A)** Nutlin-3 for 72 hours and **(B)** doxorubicin (DOXO) for 24 hours. Expression was normalized to DMSO treated cells and *GAPDH*. Results represent the mean +/- s.d. (*n* =3).

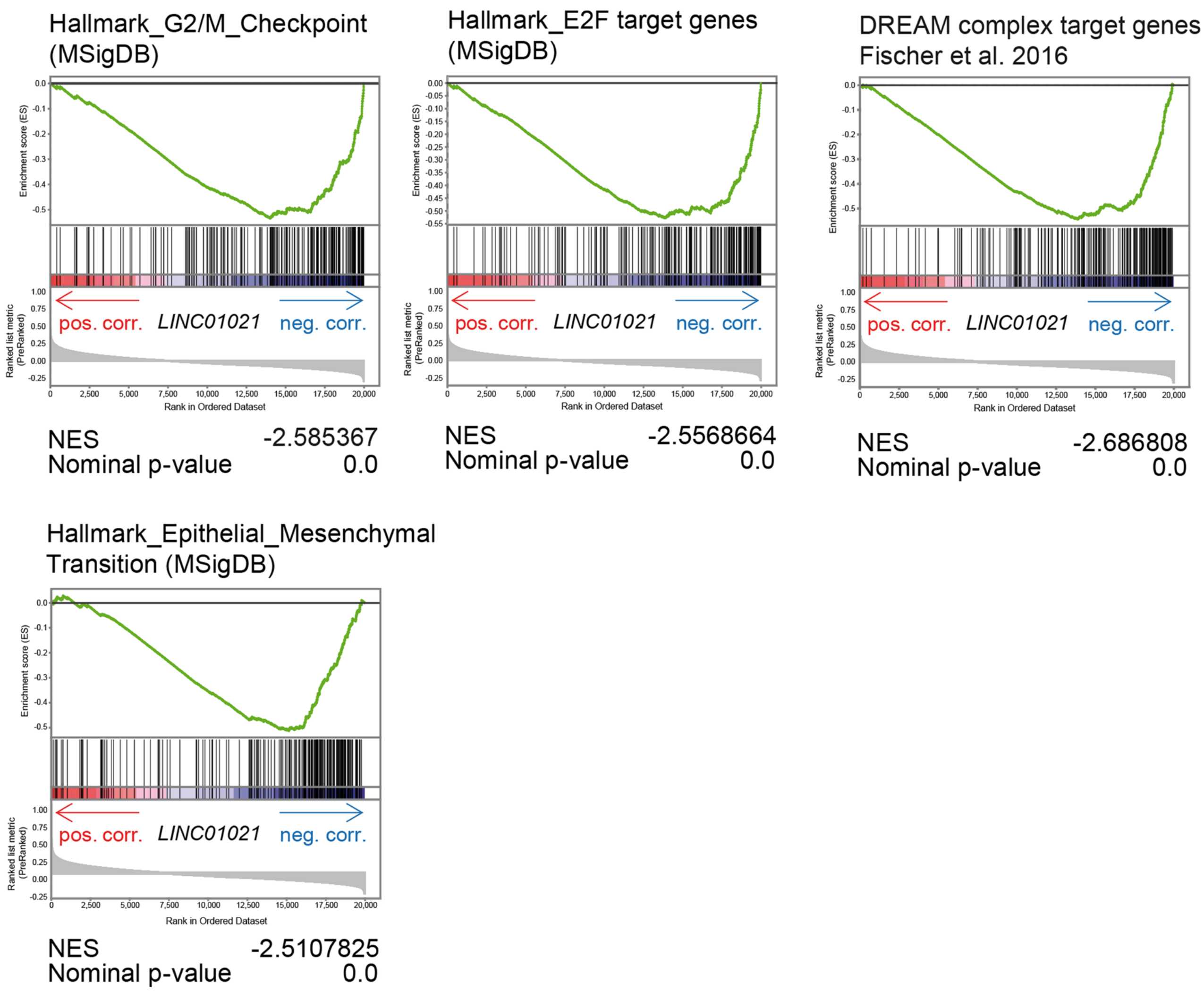

Supplementary Figure 7: Expression of *LINC01021* in colorectal tumor samples negatively correlates with the expression of genes involved in the G_2/M checkpoint, epithelial-mesenchymal transition, as well as E2F/DREAM complex target genes. The four indicated gene signatures were analyzed by GSEA. Genes were pre-ranked by expression correlation coefficient (Pearson *r*) with *LINC01021* in descending order from left (positive correlation) to right (negative correlation) based on publically available TCGA datasets from human colorectal tumors [4]. LncRNA expression data based on COAD TCGA data were obtained from [5]. Hallmark gene sets were obtained from the Molecular Signatures Database (MSigDB) [6]. The DREAM complex target gene set was obtained from [7]. Pos. corr.: positive correlation, neg. corr.: negative correlation, NES: normalized enrichment score.

Supplementary Table 1: Primers used for qPCR.

See Supplementary File 1

Supplementary Table 2: Guide RNA sequences used for CRISPR/Cas9-mediated deletions within the *LINC01021* promoter.

See Supplementary File 2

Supplementary Table 3: Primers used for cloning, mutagenesis and screening.

See Supplementary File 3
